# Scalable AC Electrospinning of Biocompatible Nanofibrous Yarns Based on Expanded Graphite and PEDOT:PSS

**DOI:** 10.3390/polym18101225

**Published:** 2026-05-17

**Authors:** Divan Coetzee, Juan Pablo Perez Aguilera, Jakub Wiener, Jiří Militký

**Affiliations:** 1 Department of Material Engineering, Faculty of Textile Engineering, Technical University of Liberec, 461 17 Liberec, Czech Republic; jakub.wiener@tul.cz (J.W.); jiri.militky@tul.cz (J.M.); 2 Department of Chemistry, Faculty of Sciences, Humanities and Education, Technical University of Liberec, 461 17 Liberec, Czech Republic; juan.pablo.perez.aguilera@tul.cz

**Keywords:** nanofibrous yarns, electrospinning, PEDOT:PSS, biocompatible, expanded graphite

## Abstract

This study presents the development of biocompatible antistatic nanofibrous composite yarns via a scalable AC electrospinning method, incorporating ultrasonicated expanded graphite (uEG) and PEDOT:PSS into polyamide (PA), polyvinyl butyral (PVB), and polyvinyl alcohol (PVA) matrices. TGA confirmed high filler retention during electrospinning. Electrical measurements showed that the addition of uEG and micrographite reduced single-yarn resistance by up to two orders of magnitude compared with neat polymers, yielding normalised resistivities as low as ~10^5^–10^6^ Ω·m and conductivities in the 10^−7^–10^−5^ S/m range, suitable for antistatic and sensing applications. However, the large filler–fibre size mismatch and highly porous yarn architecture limited the formation of continuous conductive networks, and mechanical tests revealed strength reductions of up to 70–80% at the highest PVB filler loadings. XRD confirmed a reduction in crystallinity with filler addition, PEDOT:PSS enhanced polymer chain nucleation and thus mechanical properties. Cytotoxicity assays demonstrated that uEG, micrographite, and PEDOT:PSS significantly improved cell viability compared with non-crosslinked PVA, with several PVB-based and PVA/uEG composites showing viability statistically comparable to the DMEM control (>70%) while remaining significantly higher than the Triton positive control. Overall, this work establishes an AC-electrospun route to antistatic nanofibrous yarns that combine high filler retention with enhanced biocompatibility.

## 1. Introduction

Nanofibers are characterised by their exceptionally high surface-area-to-volume ratio and unique structural properties, which can differ significantly from their microscale counterparts. Electrospinning has emerged as a versatile and scalable technique for fabricating polymer-based nanofibers, enabling the production of continuous fibres with controlled morphology and composition. The development of nanofibrous yarns—continuous assemblies of aligned or bundled nanofibers—has garnered considerable attention in materials science due to their potential to bridge the performance gap between conventional microfibre textiles and advanced functional materials [1,2,3].

Nanofiber textile structures have been demonstrated across diverse applications spanning biomedical devices, protective equipment, and advanced sensing systems. However, conventional polymer nanofibers typically exhibit limited electrical conductivity, which restricts their applicability in electrically active systems, such as wearable electronics, electromagnetic shielding, and conductive textiles [3,4,5]. The incorporation of conductive nanofiller materials has emerged as a strategic approach to enhance the electrical properties of nanofiber composites [2,6]. Carbon-based micro and nanofillers, particularly crystalline graphite or graphene and its derivatives, have demonstrated remarkable efficacy in establishing conductive percolation networks within fibre matrices, whilst being more biocompatible compared to metallic fillers [7]. Graphite is strongly anisotropic. Electrical conductivity parallel to the basal plane is about 2 × 10^5^ to 3 × 10^5^ S/m, whereas perpendicular to the basal plane it is only about 3.3 × 10^2^ S/m at 20 °C. However, integrating graphite into polymeric matrices remains challenging due to the random orientation of graphite crystals, which is the reason for the much lower fibre electrical conductivity [8].

**Figure 1:**
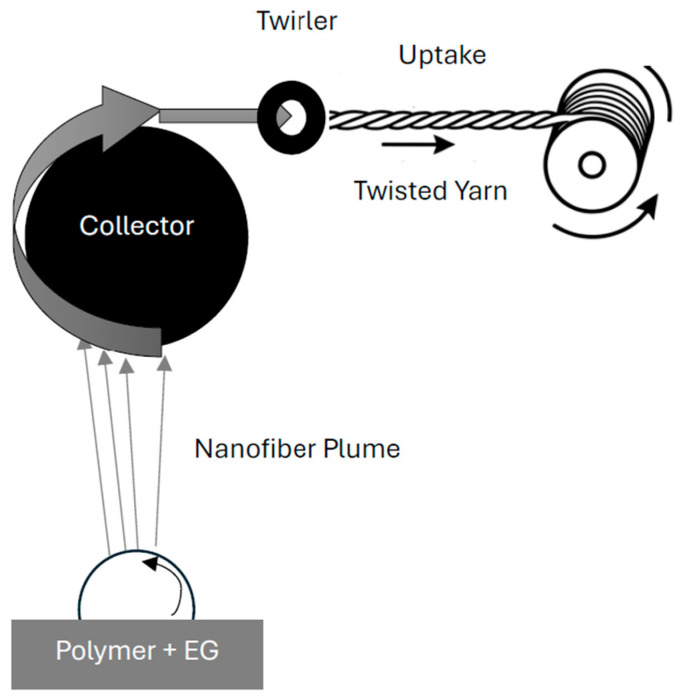
TUL patent No. 2022-370 for the production of nanofibrous yarns [10].

Nanofibrous materials are highly porous and exhibit limited mechanical properties. Yarn formation is a successful method for enhancing specific material properties by compressing the material into a cylindrical form and adding twist. Previous work has attempted this using wet laying methods followed by directional yarn collection, but these methods are difficult to scale [9]. In this work, the yarns are produced using the AC electrospinning-based technology described in TUL patent PV 2022-370 as illustrated in Figure 1, which operates in a manner analogous to open-end spinning. Nanofibers are generated from a polymer solution (or melt) on a rotating disc electrode under an alternating electric field of supercritical intensity. The resulting nanofibrous plume is transported along the electric field gradient and continuously deposited onto a rotating, electrically neutral drum collector, where it accumulates as a fluffy nanofibrous web/band. This band is then removed from the drum by a take-up twirling device; the winding unit provides the pulling force, drawing the material through the twirler, which simultaneously imparts twist. In this way, the loose nanofibrous web is transformed into a continuous, mechanically consolidated 100% nanofibrous yarn suitable for further processing [10].

EG was selected as a low-cost, carbon-based filler because graphitic carbons are widely reported to provide good electrical and thermal conductivity with generally favourable biocompatibility for biomedical and textile applications, while PEDOT:PSS was chosen as a water-processable intrinsically conductive polymer that is mechanically flexible and has been successfully used in electroconductive scaffolds and conductive fabrics [11]. EG is considered a vital step toward producing graphene because its high level of layer exfoliation provides better access to the conductive graphene planes. Ultrasonication improves this process by further exfoliating the layers and reducing particle size, making it suitable for electrospinning. Typically, expanded graphite is produced through a thermal expansion method where natural flake graphite is intercalated with oxidisers like hydrogen peroxide and acids such as sulphuric or nitric acid. Rapid heating to about 900 °C causes the material to expand up to 150 times along the c-axis of the graphene layers within the graphite structure [8]. PEDOT:PSS (poly(3,4-ethylenedioxythiophene):polystyrene sulfonate) is highly suitable as a conductive component in nanofibrous yarns because it combines intrinsic electrical conductivity with excellent processability in aqueous media. Its electrical conductivity can be improved by several orders of magnitude through secondary doping with polar solvents or by forming composites with carbonaceous fillers such as graphene or graphite, where synergistic interactions between the PEDOT chains and graphitic networks further reduce sheet resistance and enhance charge transport. In addition, PEDOT:PSS offers good mechanical flexibility, wash durability, and ambient stability on fibrous substrates [12].

For biomedical applications such as tissue-engineering scaffolds and implantable or implant-adjacent devices, carbon-based fillers like EG derivatives are particularly attractive because well-exfoliated, impurity-poor graphene nanomaterials generally exhibit good cytocompatibility at moderate doses, with cellular responses mainly governed by lateral size and residual oxidants rather than intrinsic graphene chemistry [13,14,15]. In contrast, many metal-based nanostructures (e.g., Ag, Cu, or ZnO) exhibit pronounced dose-dependent cytotoxicity driven by ion release and reactive oxygen species generation, raising concerns about long-term contact with sensitive tissues [16,17,18]. Graphene has thus proven to be a more biocompatible alternative, as clinical studies have shown that its safe dosing threshold is typically 2–10 times higher than that of common metal nanoparticles [13,14,16,18]. PEDOT:PSS further complements graphitic fillers by providing a soft, intrinsically conductive and processable phase that has been repeatedly shown to support high cell viability and even promote neural or stem-cell differentiation in electrospun or coated scaffolds, making PEDOT:PSS/graphite systems particularly well suited for biocompatible, electrically active biomedical constructs where metallic fillers may pose unacceptable toxicity and stability risks [19,20,21].

Recent advances highlight the potential of conductive fibre and yarn architectures for high-performance smart textiles. For example, Xiaohong et al. constructed electrospun liquid-metal yarns using an adhesion–channel synergy strategy that achieves high conductivity and excellent strain stability for stretchable bioelectronic interfaces. Wu et al. reported conjugated electrospun core–shell conductive nanofibre yarns integrated into fabrics for ultra-stable pressure sensing and human–machine interaction. Yin et al. have demonstrated high-strength, extensible electrospun yarns and related nanofibre architectures for energy-autonomous wearable systems, underscoring the importance of balancing mechanical robustness with electrical functionality [22,23,24,25,26]. Most current nanoyarn methods encounter issues with stability, sufficient mechanical properties, and scalability [27]. This study overcomes these challenges using a patented AC electrospinning technique. Prior studies on conductive nanofibre yarns have focused either on metallic or liquid-metal systems with exceptional conductivity but limited biocompatibility, or on graphene-based fibres prepared by complex wet-spinning or post-drawing routes. In contrast, our work combines a scalable AC electrospinning process with expanded graphite and PEDOT:PSS to produce biocompatible antistatic nanofibrous yarns directly from polymer solutions, achieving high filler retention, integrated yarn formation, and improved cytocompatibility relative to neat polymers, which distinguishes it from existing DC-electrospun mats and from most previously reported conductive yarn technologies [22].

## 2. Materials and Methods

PA 4,6 (Stanyl, Envalior, Düsseldorf, Germany) was dissolved at 14 wt% in a 1:1 mixture of glacial acetic acid and formic acid (PENTA s.r.o., Prague, Czech Republic) and magnetically stirred for 24 h. PVA (125 kDa, 89% hydrolysed, Sigma Aldrich, Darmstadt, Germany) was dissolved at 10 wt% in deionised water at 50 °C with continuous stirring, and PVB (55 kDa, Mowital, Frankfurt, Germany) was prepared as a 13 wt% solution in absolute ethanol under standard laboratory conditions. PEDOT:PSS (high conductivity grade, 3–4% aqueous dispersion, Sigma Aldrich, Germany) was added to the already prepared PVA solutions and magnetically stirred until fully incorporated. Filler-containing solutions were then obtained by dispersing uEG or micrographite via ultrasonication in the polymer solution for 10 min before electrospinning. Commercial EG (Sorbetin, Vsetín, Czech Republic) was ultrasonicated at 45 W and 40 kHz in distilled water for 1 h, until no more porous EG remained floating on the surface. Temperature control was set at 78 °C, after which the samples were removed from the ultrasonicator and cooled in an ice bath before continuing for the specified time. This was then filtered, rinsed with distilled water to neutralise and remove excess peroxide, and dried in an oven at 80 °C for 30 min. Micrographite (mGraph; Epinikon a.s., Vodňany, Czech Republic) is a commercial synthetic micro-pulverised graphite powder composed of flat, irregular platelet-like particles (≥99.0% C) with particle sizes between 3.5 and 5.0 μm, as specified by the manufacturer. In this work, mGraph was used as a non-expanded, micrometre-scale graphitic filler that was dispersed directly into PVB solutions via ultrasonication and electrospun into nanofibrous yarns; it was not intercalated or expanded together with uEG. Electrospinning parameters were as follows: electrode disc size (200 mm); voltage: 35 kV; 50 Hz sinusoidal waveform; twirler eye: double eyelet (5 and 6.2 eccentricity); twirler rpm: 9000; winding speed: 1 m/min; and 1.8 m/min for PVB 20% uEG fast sample. All were spun on an AC electrospinning machine adapted for nanofibrous yarn production (Technical University of Liberec, Liberec, Czech Republic).

Samples were characterised using a scanning electron microscope (SEM, Vega 3 Tescan, Brno, Czech Republic). Electrical measurements were performed according to ASTM D4496 standard using a Hewlett-Packard 4339B (Korea) with yarns arranged in parallel (*n* = 48) over 1 cm between two electrodes at 24 °C and 42% RH [28]. Tensile tests were conducted on an Instron 3400 tensile tester (*n* = 10) (Norwood, MA, USA). Thermogravimetric analysis (TGA; Mettler Toledo, Giessen, Germany) was performed (*n* = 1) to determine the filler content of the spun materials. Fourier-transform infrared (FTIR) spectra of the PA, PVA, and PVB samples and their composites were recorded using an FTIR spectrometer, Nicolet iZ10 (Thermo Scientific, Waltham, MA, USA), equipped with a single-reflection ATR accessory. Measurements were performed in the mid-infrared region from 4000 to 400 cm^−1^, with a spectral resolution of 4 cm^−1^ and averaging 32 scans per spectrum in absorbance mode. The nanofibrous yarns were placed directly onto the ATR crystal with gentle pressure applied to ensure good optical contact. The resulting spectra were normalised at a characteristic C–H stretching band to enable comparison between neat polymers and filled samples, and peak positions were assigned based on literature data. XRD analysis was performed using a Philips Panalytical PRO MPD (Netherlands) with a copper source and a PIXcel 1D detector. The initial and final 2-theta angles were 5° and 90°, respectively, with a step size of 0.017°. Cytotoxicity was assessed using an extract-based method in accordance with ISO 10993-5:2009, employing L929 mouse fibroblasts as the test cell line. Yarn samples, each 1 m in length, were cut, weighed, and extracted in Dulbecco’s modified Eagle Medium (DMEM) at an extraction ratio of 10 mg mL^−1^ for 24 h at 37 °C. The resulting extracts were applied to L929 cell monolayers at 100 µL per well in well plates (*n* = 8 wells per condition), with DMEM alone as the negative control and 1% Triton X-100 in DMEM as the positive control. Cell metabolic activity after 24 h exposure was quantified using a water-soluble tetrazolium-salt assay (CCK-8), which provides results comparable to conventional MTT assays for assessing material cytotoxicity. Viability was normalised to the negative control and values ≥ 70% were considered non-cytotoxic, following ISO 10993-5 criteria. Swelling behaviour and in vitro mass loss of nanofibrous yarns were evaluated by immersion in phosphate-buffered saline (PBS, pH 7.4; Sigma-Aldrich, St. Louis, MO, USA) at 37 °C for 7 days, following established protocols for nanofibrous biomaterials. Yarn samples of fixed length (10 cm) were dried to constant weight in a thermostatted oven (Binder APT.line, Binder GmbH, Tuttlingen, Germany) at 40 °C and weighed to obtain the initial dry mass (W_0_). Each sample was transferred to a 1.5 mL polypropylene microcentrifuge tube (Eppendorf AG, Hamburg, Germany) containing 1.5 mL PBS and incubated in a thermostatted orbital shaker incubator (ES-20, Biosan, Riga, Latvia) at 37 °C and 100 rpm. After 7 days, samples were carefully removed using sterilised tweezers, gently blotted with filter paper to remove surface liquid without compression, and immediately weighed to obtain the swollen mass (W_s_). The swelling ratio was calculated as [(W_s_ − W_0_)/W_0_] × 100. Samples were then re-dried to constant mass (W_d) under the same conditions, and mass loss was determined as [(W_0_ − W_d)/W_0_] × 100. Measurements were performed in triplicate (*n* = 3).

## 3. Results and Discussion

### 3.1. Microscopic Analysis

**Figure 2:**
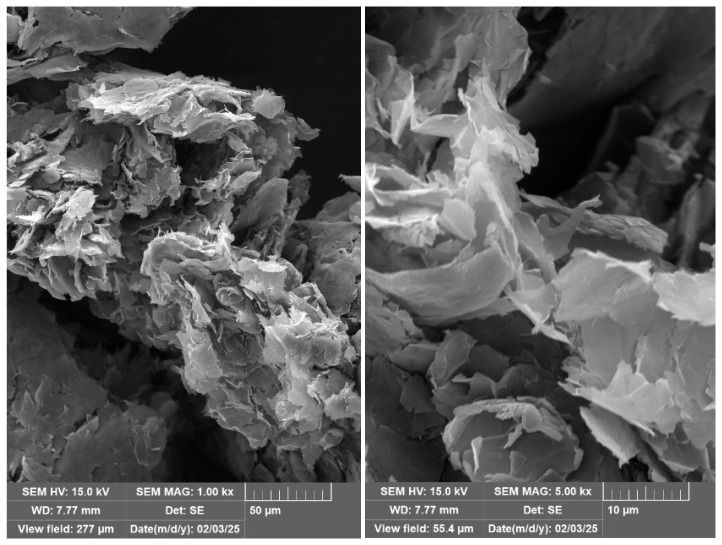
SEM of ultrasonicated expanded graphite at 1000× magnification (**left**) and 5000x magnification (**right**).

Dry ultrasonicated expanded graphite tends to form agglomerates after filtration, as shown in the SEM images of the graphene-like flakes in Figure 2. These images clearly demonstrate that while the graphene or thin-layered graphitic sheets are within the nanometre scale, their in-plane particle size can extend to the micrometre range.

**Figure 3:**
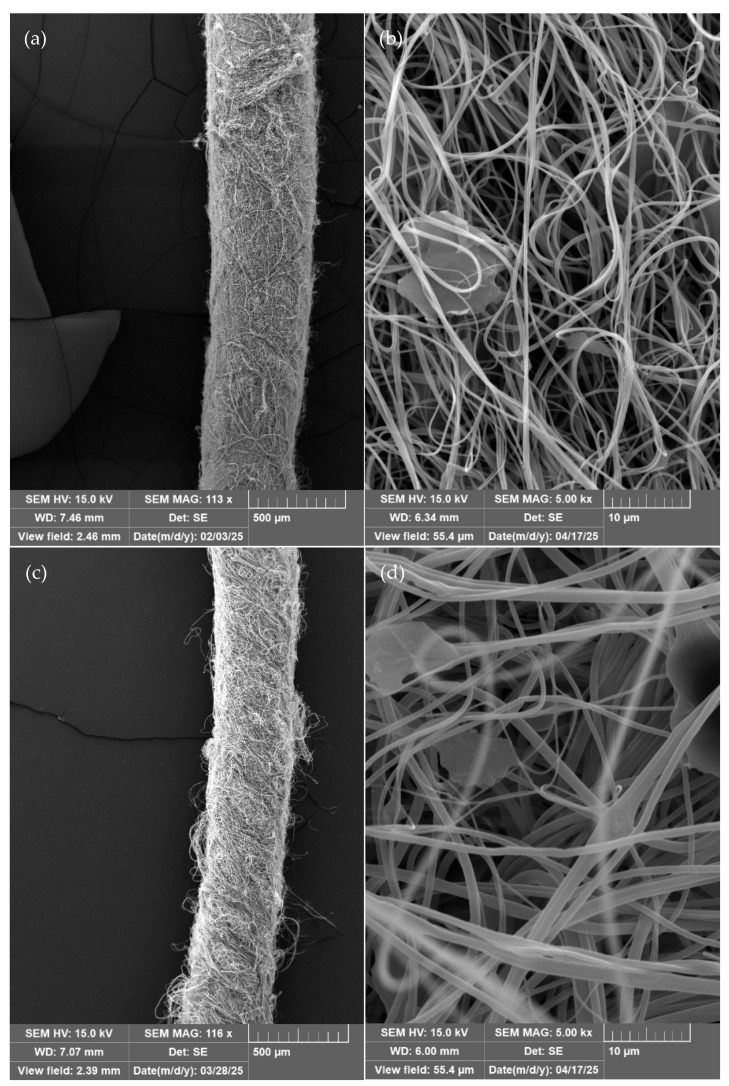

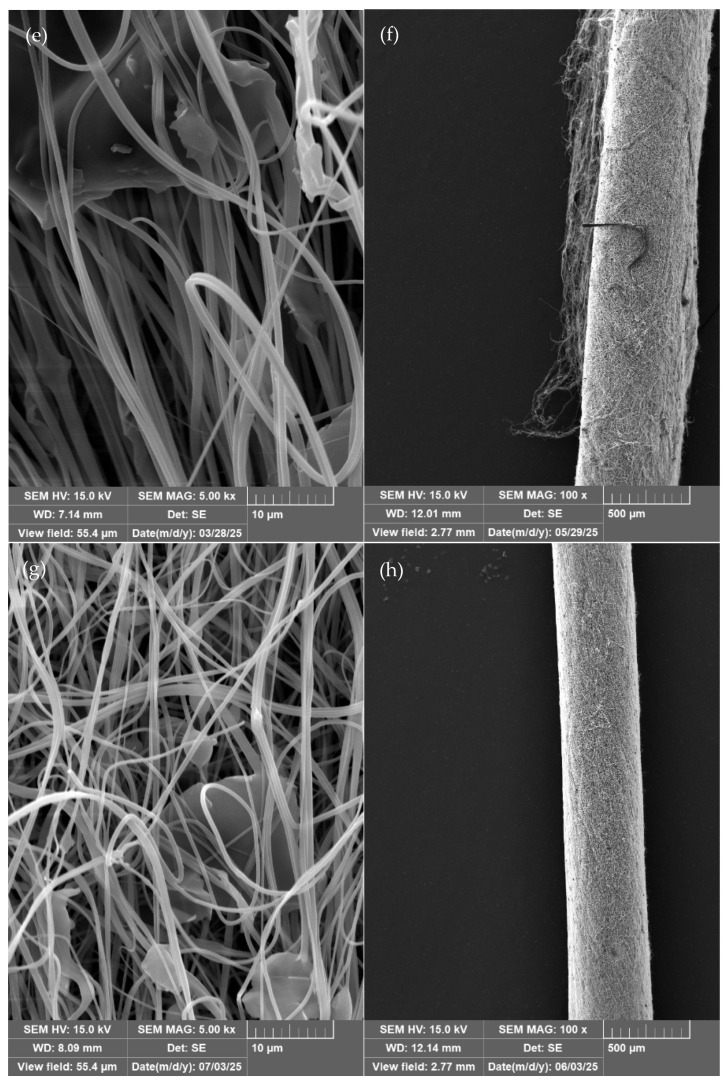

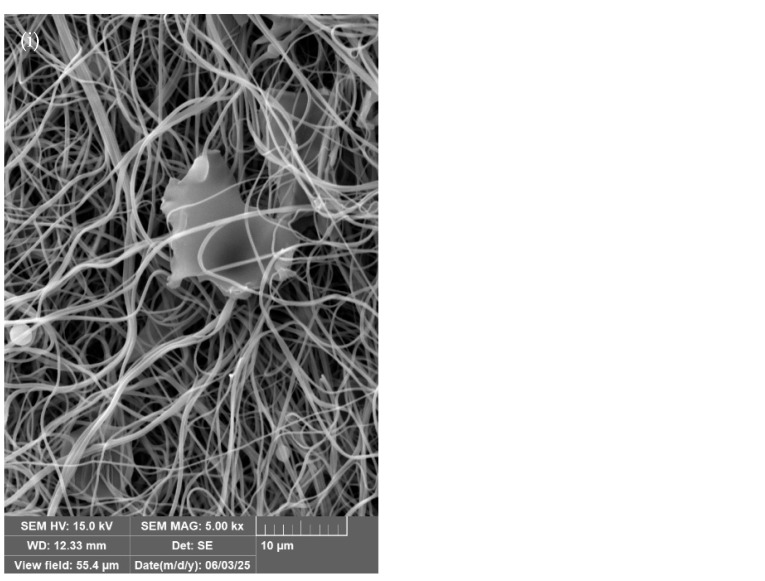
SEM of nanofibrous yarns—(**a**) PVA; (**b**) magnified image of PA yarn containing 10% uEG; (**c**) PVB; (**d**) magnified image of PVB containing 30% mGraph; (**e**) magnified image of PVB containing 20% uEG; (**f**) PVA; (**g**) PVA containing 10% uEG; (**h**) PVA with PEDOT:PSS; (**i**) PVA:PEDOT:PSS with 10% uEG.

Quantitative image assessment confirmed that uEG flakes typically span several micrometres in the in-plane direction, whereas the electrospun fibres exhibit sub-micrometre diameters (Figure 3). This large size mismatch means that many flakes intersect multiple fibres and can only be partially embedded rather than fully encapsulated, disrupting fibre continuity and favouring particle–particle contacts at the yarn surface rather than within a continuous network within individual nanofibres. Similar behaviour has been reported for expanded-graphite or graphene–nanoplatelet fillers in electrospun composites, where insufficient lateral size reduction leads to high percolation thresholds and limited conductivity improvements in highly porous fibrous architectures [22,29].

There is visual evidence of particle–particle contact; however, the structure appears to be dominated by yarn porosity, which is expected to reduce the electrical conductivity of the samples. For the samples containing the uEG particles, it was noted that the particles in plane dimensions were in the micrometre range, whereas the c-axis direction exhibited nanometre-scale thickness with a few-layer graphene structure. Due to the large size difference between the particles and the fibres, the particles could not be fully incorporated into the fibres, as only partial encapsulation occurred. Samples containing micrographite were larger in both axes and, therefore, are expected to have a higher percolation threshold compared to the ultrasonicated graphite.

The effect of filler loading on AC electrospinning jet stability was quantitatively assessed through nanofiber diameter distributions as illustrated in Figure 4 (*n* = 100 fibres/sample, Supplementary File Figures S1–S15, Table S1), using uniformity metrics alongside SEM morphology as complementary indicators of process robustness. All formulations produced continuous sub-micrometre nanofibres (mean diameters 351–1039 nm, CV% 30.7–97.1%) with filler-specific effects that varied strongly by polymer matrix, revealing distinct mechanistic responses.

PA 4,6 systems exhibited intrinsic robustness, maintaining fine diameters (422.1 ± 129.4 nm → 350.5 ± 123.4 nm, CV% 30.7–35.2%, IQR 132–148 nm, 1–6% outliers) across uEG loadings. The modest diameter refinement without increased diameter variability indicates uEG enhanced charge dissipation and jet elongation without rheological disruption, confirming PA’s tolerance for graphitic fillers under fixed AC parameters. PVA systems demonstrated sharp additive specificity. Neat PVA produced unstable fibres (471.0 ± 337.8 nm, CV% 71.7%, IQR 225 nm, 8% outliers) with SEM-confirmed beading, reflecting poor jet control in the non-crosslinked aqueous system. Critically, PVA 10% uEG transformed this into uniform, bead-free nanofibres (466.4 ± 158.7 nm, CV% 34.0%, IQR 158 nm), demonstrating uEG’s multifunctional stabilisation through conductivity-mediated jet stretching and worm-like flake entanglement suppressing Rayleigh breakup. Conversely, PEDOT:PSS variants exacerbated instability (CV% 54.8–97.1%, PVA 15/15%: 550.7 ± 534.5 nm, 12% outliers) with persistent beading, as polyelectrolyte plasticisation overwhelmed charge-transport benefits. PVB systems revealed systematic loading control. Neat PVB yielded coarser fibres (1038.8 ± 526.4 nm, CV% 50.7%, IQR 682 nm) due to high solution viscosity, while mGraphite loaded progressively refined morphology (10%: 877.0 nm → 20%: 694.8 nm → 30%: 874.8 ± 316.4 nm, CV% 36.2–44.8%). The optimal PVB 30% mGraphite achieved zero outliers and tightest distribution (IQR 488 nm) correlating with its superior electrical performance, while uEG variants maintained moderate diameter variability (CV% 32.5–49.6%, PVB 20% uEG fast: 665.8 ± 216.2 nm). No PVB samples showed beading, indicating fillers modulated diameter through viscoelastic balance rather than morphological defects [30].

These polymer-specific responses establish clear design rules: PA tolerated fillers robustly through inherent jet stability; PVA required chemically specific stabilisation (uEG effective, PEDOT:PSS destabilising); and PVB enabled loading-dependent control, maximising uniformity at high micrographite content. The quantitative metrics directly correlate fibre uniformity with downstream yarn electrical performance, validating AC electrospinning as a versatile platform for antistatic nanofibrous composites.

### 3.2. TGA Analysis

TGA of the produced nanofibrous yarns indicated good filler retention during production. The TGA results as residual carbonaceous material are presented in Table 1. For PA and PVA, the residue values were determined at 780 °C, and for PVB samples at 760 °C after the plots exhibited a single-step degradation profile with no distinctive features (Supplementary File Figures S16–S18).

The thermogravimetric curves of all yarns exhibited a small initial mass loss between 50 and 100 °C, corresponding to about 3–5% for PVA, 1–1.5 wt% for PA and <1% for PVB, followed by a predominant single-step mass loss associated with polymer backbone degradation. This low-temperature loss is attributed mainly to desorption of physically adsorbed moisture and loosely bound water in the nanofibrous structures, which is most pronounced for the hydrophilic PVA yarns and very limited for the more hydrophobic PA and PVB. Above ~100–120 °C, the curves show no additional distinct shoulders that would indicate significant amounts of retained processing solvents, and the onset and peak decomposition temperatures are characteristic of PA, PVA and PVB. Any residual water, ethanol, acetic acid, or formic acid introduced during electrospinning is therefore expected to evaporate during post-spinning drying and the initial heating stage of TGA; any remaining signal may be obscured by water loss below 200 °C and is expected to contribute negligibly to the residual mass measured at 760–780 °C.

The carbonaceous residue contribution from fillers was calculated as the difference between the % residue of filled samples and their respective neat or baseline polymer, as presented in Table 1. For PVA systems containing both PEDOT:PSS and uEG, the baseline was the PVA 10% PEDOT:PSS sample (2.98%) rather than neat PVA (5.11%), reflecting the modified polymer matrix. The PVA 10% PEDOT:PSS sample exhibits a slightly lower char residue (2.98%) than neat PVA (5.11%). In PVA/sulfonated-polyelectrolyte systems such as crosslinked PVA/PAMPS membranes, increasing the sulfonated polymer content has been reported to lower thermal stability and reduce the residual mass at high temperature compared with PVA alone, indicating that introducing an acidic, highly labile component does not necessarily increase char yield. In our case, PEDOT:PSS is present at low loading, is fully organic, and is finely dispersed within the PVA network, so it contributes little inorganic residue; the modest reduction in overall char fraction for PVA 10% PEDOT:PSS relative to neat PVA is therefore consistent with the specific composition and degradation pathway of this composite [31,32]. Overall, the filler content in the produced sample was lower than that in the prepared solutions, attributed to incomplete incorporation during the electrospinning process used for yarn production.

Strong positive correlations were observed between nominal filler weight percentage and total% residue for PA (r = 0.957) and PVB (r = 0.871), indicating effective filler incorporation during spinning for these polymers for both uEG and mGraph. PA 10% uEG yielded 9.90% residue (8.33% carbon gain vs. neat PA), while PVB systems showed exceptional performance with mGraph: 32.94% residue at 30 wt% (29.66% carbon gain vs. neat PVB). PVB uEG systems averaged 7.96% carbon gain across 10–20 wt% loadings, though processing conditions significantly influenced retention: PVB 20% uEG “fast” achieved 9.69% carbon gain versus 7.38% for “slow” (both vs. neat PVB), suggesting the faster take-up rate resulted in improved filler incorporation. PVA systems exhibited formulation-dependent behaviour. While 10% uEG alone produced a 5.20% carbon gain compared with neat PVA, 10% PEDOT:PSS reduced the residue to 2.98%. Combined systems showed partial recovery: PVA 10% PEDOT:PSS with 10% uEG gave 4.22% uEG gain (vs. PEDOT baseline), and 15% PEDOT:PSS + 15% uEG yielded 8.52% uEG gain (vs. same baseline). Overall, PVB + mGraph demonstrated the highest degree of filler retention during electrospinning, followed by PVB + uEG and PA + uEG, with PVA systems requiring optimised filler combinations to maximise filler incorporation.

### 3.3. FTIR Analysis

**Figure 5:**
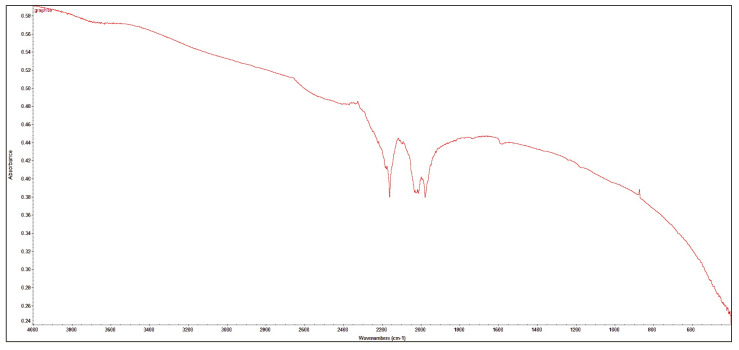
FTIR of ultrasonicated expanded graphite.

FTIR absorbance measurements of the uEG (Figure 5) showed additional weak bands at approximately 2160 cm^−1^ and a doublet near 2050 and 1980 cm^−1^. These lie in the region typically associated with triple-bond or pseudo-triple-bond stretching modes (e.g., C≡N, C≡C, or related N-containing groups) and combination/overtone bands, and similar low-intensity features around 2160 and 1960 cm^−1^ have been reported for oxidised graphite prepared in mixed-acid systems and attributed to nitrogen-containing functionalities introduced during oxidative intercalation. In this case, the very low intensity of these bands, together with the absence of strong additional carbonyl/carboxyl peaks and the dominance of the usual oxygen-containing groups observed for mildly oxidised graphitic carbons (broad O–H around 3200–3500 cm^−1^, C=O near 1700–1730 cm^−1^, and C–O/C–O–C modes between about 1000–1300 cm^−1^), indicates that the intercalation–expansion–ultrasonication sequence leads primarily to moderate oxidation at edge and defect sites rather than to the heavily functionalised graphene-oxide-type structure. Overall, the FTIR results therefore support the fact that uEG retains a largely graphitic framework with a limited population of oxygenated and possibly nitrogen-containing defect sites, which is sufficient to enhance wettability and dispersion in polar polymer matrices while preserving its conductive character [33,34].

FTIR was used to verify the chemical integrity of the PA, PVA and PVB matrices and composites to probe possible interactions with the carbonaceous fillers (Supplementary Figures S19–S21). The spectra of the neat polymers showed the expected characteristic bands: PA displays a broad N–H stretching band at ~3290 cm^−1^ and amide I/II bands at ~1633 and ~1535 cm^−1^, respectively; PVA exhibits a broad O–H stretching band at ~3288 cm^−1^, C–H stretching at ~2912 cm^−1^ and a strong C–O stretching band near 1085–1090 cm^−1^; PVB shows C–H stretching around 2950–2935 cm^−1^, combined CH_2_/CH_3_/OH bands at ~1430–1380 cm^−1^, and intense acetal C–O–C/C–O bands at ~1130–1105 cm^−1^ in agreement with literature assignments. Upon incorporation of uEG, micrographite or PEDOT:PSS, no new absorption bands appeared and the positions of the main polymer bands remained essentially unchanged; only modest changes in intensity and broadening of the N–H/O–H stretching envelopes and selected fingerprint bands were observed, consistent with physical interactions (e.g., hydrogen bonding or adsorption at filler surfaces) rather than formation of new covalent bonds. The absence of additional carbonyl, aromatic or other new vibrational signatures indicates that the processing conditions used did not induce chemical reactions between the polymers and the fillers, and that the fillers were dispersed in the matrices without altering the backbone chemistry.

### 3.4. Electrical Measurements

The electrical resistivity data (Table 2) show that the addition of uEG and micrographite substantially reduced resistivity relative to neat polymer yarns, but the resulting properties remain in the antistatic/electrostatically dissipative regime rather than in the range required for bulk conductive conductors. Control yarns devoid of conductive fillers exhibit single-yarn resistances on the order of 10^13^ Ω, whereas the highest-performing PVB 30% mGraph and PVA 15% PEDOT:PSS 15% uEG formulations reduce single-yarn resistances to ~10^11^ Ω and normalised resistivities down to ~10^5^–10^6^ Ω·m, which are appropriate for antistatic and sensing applications but below the performance of metallic or highly percolated carbon systems [22]. When normalised by the linear weight of the samples, the conductive difference becomes more pronounced as fibre fineness improves. Insulative polymers exhibit resistivities as high as 2.16 × 10^8^ Ω·m/tex with the addition of expanded graphite and PEDOT:PPS, reducing the normalised resistivity to 1.37 × 10^5^ Ω·m/tex.

AC electrospinning yields a three-dimensional, porous yarn structure, which may limit conductivity relative to the denser films predominantly discussed in the existing literature. Nonetheless, recent reviews contend that electrospun yarns can sustain adequate flexibility and mechanical integrity while attaining electrical properties suitable for antistatic and smart textile applications, contingent upon well-dispersed fillers and sufficient filler content. Conductivity values for yarns incorporating PEDOT:PSS have been documented within the range of 10^−2^–10^4^ S/m, corresponding to resistivities spanning 1–10^6^ Ω·m, with performance heavily influenced by post-processing and secondary fillers. This study demonstrates that PEDOT:PSS uEG composite yarns approach these conductivity levels; however, additional post-treatment is required. Metallic fillers continue to provide superior conductivity enhancements, as demonstrated by the comparative literature [21,35,36,37].

Compared with conventional DC-electrospun nonwoven mats of similar composition, the AC-spun yarns produced here exhibit a more three-dimensional, twisted architecture with higher porosity and lower packing density, which is advantageous for flexibility and textile processing but generally yields lower effective conductivity. DC-electrospun mats collected as dense webs or after hot-pressing can achieve lower percolation thresholds and higher conductivities due to more continuous particle networks and reduced interfibre voids, as reported for expanded-graphite- and graphene-based nonwovens [22]. In contrast, the AC-spun yarns in this work prioritise scalability and yarn formability, so their performance currently aligns with antistatic and sensing applications rather than high-current EMI shielding, but further densification and particle size reduction could narrow this gap. The present work used a fixed twist level and no post-calendering, so the yarn structure retained a relatively high porosity, which limits fibre–fibre contacts and percolation. In conductive yarn systems, mechanical densification and post-drawing have been shown to improve fibre packing and electrical connectivity [38]. Future work could therefore focus on systematic twist optimisation and post-drawing or calendering treatments to translate the demonstrated filler efficacy into denser and possibly more conductive yarn architectures.

The literature on uEG/polymer fibres indicates that effective exfoliation and dispersion can yield percolation thresholds as low as 7–10 vol%, with resistivities below 10^8^ Ω·m at optimal loadings in denser fibre configurations, such as compressed films [39,40]. The lower electrical resistivities of the yarns are attributed to the porosity and partial particle encapsulation by the polymer during spinning, both causing insulating effects. Micrographite fillers demonstrate lower intrinsic conductivity and larger particle sizes, resulting in higher percolation thresholds and a more gradual improvement in conductivity with increasing content. This is attributed to the anisotropic electrical conductivity of graphite. Comparative investigations reveal that micrographite composites exhibit percolation thresholds within the range of 15–25 vol% and resistivities in the domain of 10^8^–10^10^ Ω·m after reaching critical loading. The nanofibrous yarns produced in this research are consistent with literature reports for analogous fibrous structures, where further enhancements depend on exfoliated graphite fillers such as nanoplatelets [39,41,42].

### 3.5. Tensile Measurements

The tensile measurement of the nanofibrous yarns is presented in Table 3. Overall, the addition of uEG and micrographite resulted in a reduction in mechanical properties in all samples. This was attributed to the microscale dimensions of the fillers, resulting in weaker filler incorporation into the fibres as the size difference creates a weaker filler–polymer interface. Improvements can be achieved through techniques such as ball milling to further reduce particle sizes and better align with the nanoscale of the individual fibres in the yarns. This is also expected to enhance the formation of conductive pathways within each fibre, overcoming the limitations experienced in this work.

**Figure 6:**
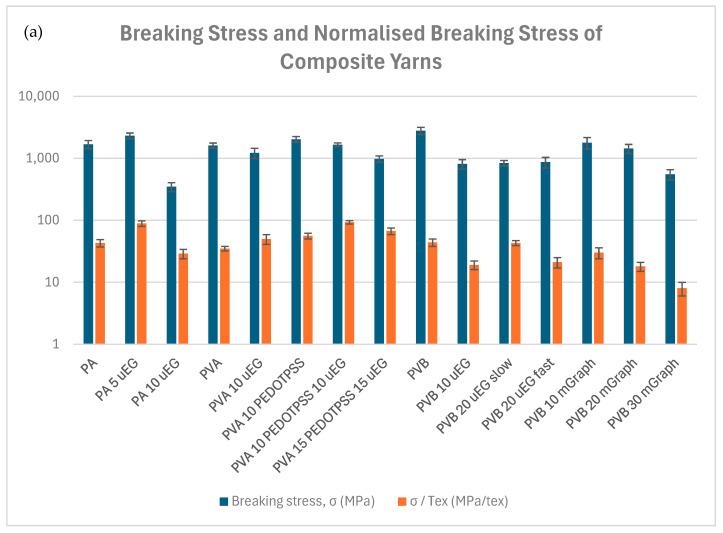

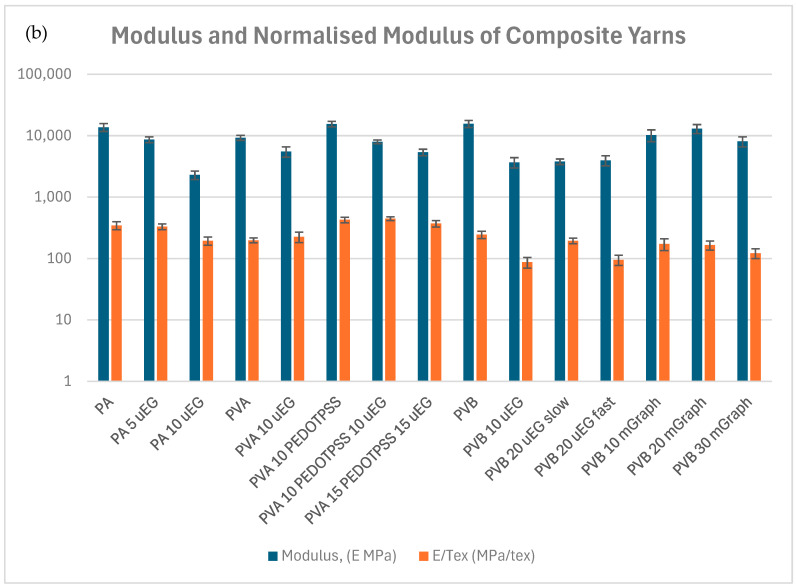
Breaking stress and normalised breaking stress (MPa·tex) (**a**) and tensile modulus and normalised modulus (MPa·tex) (**b**) of nanofibrous yarns based on PA, PVA and PVB with varying uEG, PEDOT:PSS and micrographite contents (all formulations listed in Table 3; mean ± SD, *n* = 10).

To visualise the effect of filler type and loading on yarn mechanics, Figure 6 compares breaking stress, normalised strength and modulus for all formulations listed in Table 3. Overall, the trends show that mechanical performance depends strongly on both filler type and concentration, with the most pronounced reductions occurring in the PVB systems at higher micrographite loadings. In these samples, the larger filler size and less favourable filler–matrix compatibility appear to limit stress transfer and promote defect formation, which leads to a clear decline in strength. By contrast, the PA and PVA systems show more moderate responses, and intermediate filler additions can be accommodated more effectively without such severe losses in mechanical integrity. This indicates that the mechanical behaviour of the yarns is governed by a balance between filler incorporation, dispersion quality and the intrinsic characteristics of each polymer matrix.

The normalised metrics (σ/Tex, E/Tex) show more nuanced effects, with PA and PVA exhibiting paradoxical increases in some cases, likely due to reduced yarn tex (linear density) from filler incorporation or processing changes. Average relative changes vs. neat polymers are as follows: PA (σ/Tex +25 ± 73%, E/Tex −16 ± 24%); PVA (σ/Tex +72 ± 62%, E/Tex +68 ± 58%); PVB (σ/Tex −41 ± 31%, E/Tex −37 ± 23%).

Unfilled PA yarns exhibit balanced properties with σ = 1689 MPa, E = 13.7 GPa, and elongation ~30%, typical for semicrystalline polyamides. Adding 5 wt% uEG boosts normalised strength (σ/Tex from 43 to 89 MPa/tex, +107%) despite halving breaking force (1.08 → 0.68 N), suggesting thinner yarns with preserved or enhanced stress at break per mass. However, 10 wt% uEG severely degrades all metrics—breaking force drops to 0.18 N (−83%), σ/Tex falls to 29 MPa/tex (−33%), and E/Tex to 194 MPa/tex (−44%)—indicating poor interfacial adhesion and defect introduction from large flakes disrupting fibre continuity, as visually confirmed by SEM. PVA shows moderate strength (σ = 1614 MPa, σ/Tex = 35 MPa/tex) but low elongation (14.6%). uEG at 10 wt% reduces breaking force (0.73 → 0.43 N) and σ (to 1223 MPa) but raises σ/Tex (+43%) and dramatically increases elongation to 64.2% (+340%), possibly from plasticising effects or altered web compaction during AC spinning. PEDOT:PSS alone (10 wt%) improves breaking force (+67%, 1.22 N) and E/Tex (+115%, 427 MPa/tex), highlighting its compatibility as a soft conductive dopant. Synergistic PEDOT:PSS + uEG (10/10 or 15/15 wt%) maintains elevated σ/Tex (67–93 MPa/tex, +91–166% vs. neat) but lowers elongation and modulus, underscoring a strength–ductility trade-off from filler agglomeration. PVB excels with the highest σ (2798 MPa) and E (15.6 GPa), but fillers uniformly impair performance. Both uEG (10–20 wt%) and mGraph (10–30 wt%) slash breaking force by 75–87% (e.g., 1.23 → 0.29–0.63 N), σ by 70–80% (to 552–1786 MPa), and σ/Tex by 57–82% (to 8–30 MPa/tex), with E/Tex dropping 55–65% (87–194 MPa/tex). Higher loadings worsen effects (PVB 30% mGraph: σ/Tex = 8 MPa/tex, −82%), and processing speed minimally influences uEG samples. This polymer appears most sensitive to filler size mismatch, likely due to its brittle nature, amplifying crack propagation at flake interfaces.

The pronounced 70–80% reductions in breaking force and σ/Tex for PVB composites can be attributed to the combination of high filler loading and the glassy character of PVB at room temperature, which makes it more prone to brittle failure than the more ductile PA and PVA matrices under our test conditions. Micrometre-scale graphite flakes act as stress concentrators and promote interfacial debonding under tensile load, especially when aggregated or only partially embedded in the nanofibres, which accelerates crack initiation and propagation along particle-rich regions. Similar degradations in strength at high graphite or graphene–nanoplatelet contents have been reported for other glassy or semicrystalline polymer matrices, where conductivity and stiffness gains are achieved at the expense of tensile strength and ductility [43,44,45,46]. Only quasistatic tensile tests were conducted in this study, so viscoelastic behaviour and glass-transition shifts could not be evaluated directly. Dynamic mechanical analysis (DMA) on neat and filled yarns would provide complementary insight into filler–matrix interactions through changes in storage modulus, damping behaviour, and potential *T_g_* shifts, and will therefore be pursued in future work to extend the present mechanical assessment.

The strength and modulus values obtained in this study fall within the broad range reported for electrospun nanofiber yarns, which typically show tensile strengths from a few tens up to several hundred megapascals and moduli on the order of 0.5–10 GPa, depending on polymer, twist, and post-drawing. For example, hot-stretched PAN nanoyarns have achieved breaking stresses of 146–372 MPa with moduli up to ~11.8 GPa, and recent high-strength PAN nanofiber yarns functionalised with silver nanowires reached a tensile strength of ~743 MPa with a modulus of ~12 GPa. In this context, our neat PA and PVB nanofibrous yarns, which exhibit strengths in the gigapascal range and moduli around 13–16 GPa when calculated from the SEM-derived cross-sectional areas, lie at the upper end of the reported performance envelope for electrospun yarns, while the heavily filled composites show the expected reduction in strength and stiffness, highlighting the trade-off between electrical enhancement and mechanical robustness [27,47,48].

### 3.6. XRD Analysis

**Figure 7:**
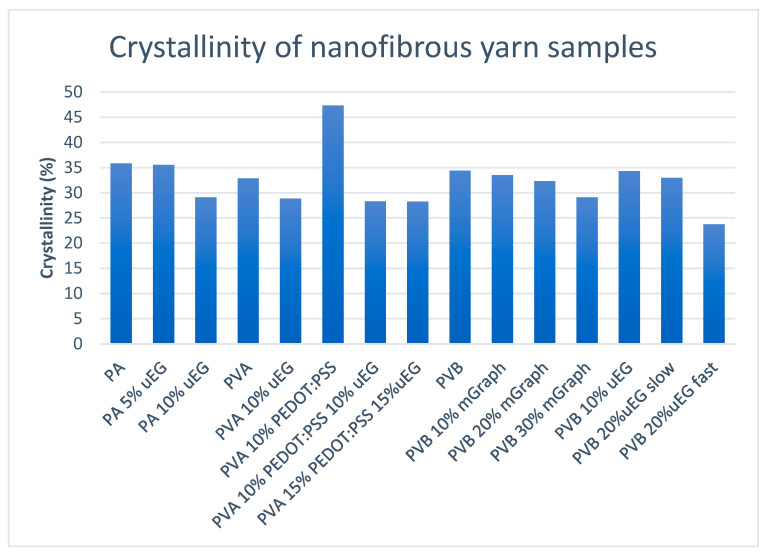
Crystallinity of nanofibrous yarn samples as determined using XRD.

Wide-angle X-ray diffraction was used to quantify the degree of crystallinity of the nanofibrous yarns by deconvoluting the crystalline peaks and amorphous halo and integrating their respective areas for each formulation, as illustrated in Figure 7 (individual sample diffraction spectra are available in the Supplementary Figures S22–S36). The resulting crystallinity indices, calculated as the ratio of crystalline peak area to total scattering area over the selected 2θ range, lie between approximately 24 and 47% across all systems, indicating that the AC-electrospun yarns are semicrystalline rather than highly crystalline. These values are lower than those reported for bulk PA 4,6, which can reach crystallinities on the order of 60–70%, and also lower than typical injection-moulded PVA or PVB, reflecting the formation of smaller and more defective crystals in electrospun nanofibres, as widely observed for electrospun polymers. This reduction in crystallinity is commonly attributed to rapid jet stretching and solidification during electrospinning, which promotes chain orientation but limits the time available for growth of well-developed lamellae [49,50].

For the PA 4,6-based yarns, the crystallinity index of the neat polymer is about 35.8% and remains essentially unchanged at 35.6% upon addition of 5% uEG, before decreasing to 29.1% at 10% uEG. The minimal change at low filler loading indicates that small amounts of uEG flakes do not strongly disturb the semicrystalline morphology of PA 4,6, which is consistent with the known high crystallisation tendency and hydrogen-bonded network of this polymer. The reduction in crystallinity at 10% uEG suggests that higher filler contents begin to interfere with chain packing and crystal growth, likely due to the micrometre-scale lateral dimensions of the uEG flakes and their partial encapsulation within the nanofibres, as seen by SEM. However, the tensile data show that breaking stress and modulus drop much more dramatically between PA and PA 10% uEG (from 1689 to 349 MPa and from 13.7 to 2.3 GPa, respectively), whereas PA 5% uEG retains relatively high modulus and a higher normalised strength. This indicates that the severe mechanical degradation at 10% uEG is governed primarily by interfacial defects and stress concentrations introduced by large, poorly embedded flakes, with the modest crystallinity reduction acting as a secondary factor, in line with reports on PA/expanded-graphite or graphene–nanoplatelet composites where high filler loadings and size mismatch weaken the fibre network despite only moderate changes in crystallinity [51,52].

In the PVA systems, neat PVA nanofibrous yarns show a crystallinity of 32.9%, which decreases to 28.8% when 10% uEG is incorporated. This trend is consistent with studies on electrospun PVA nanofibres and PVA nanocomposites, where modest amounts of nanoscale fillers can nucleate crystals, but higher loadings or larger particles disrupt chain orientation and produce smaller, more defective crystallites with lower overall crystallinity. Interestingly, the PVA 10 PEDOT:PSS sample exhibits a substantially higher crystallinity index of 47.3%, suggesting that PEDOT:PSS at this loading promotes additional ordering, either by acting as a nucleating phase or by inducing more efficient packing of PVA chains and PEDOT nanodomains, as has been reported for PEDOT:PSS/PVA thin films and related composites where conductive domains drive more ordered structures [53]. When uEG is added together with PEDOT:PSS (PVA 10% PEDOT:PSS 10% uEG and PVA 15% PEDOT:PSS 15% uEG), the crystallinity drops back to ~28.3%, indicating that the disruption introduced by the relatively large graphitic flakes outweighs any nucleating effect from PEDOT:PSS [49,50,53]. These crystallinity trends correlate well with the tensile behaviour of the PVA yarns. Neat PVA shows moderate strength (1614 MPa) and low elongation (14.6%), while PVA 10 uEG exhibits reduced strength (1223 MPa) but markedly increased elongation (64.2%), consistent with a slightly lower crystallinity and a more defect-rich, ductile network. In contrast, PVA 10 PEDOT:PSS, which has the highest crystallinity (47.3%), displays the highest breaking stress (2031 MPa) and modulus (15.5 GPa), pointing to a more ordered and load-bearing structure. At higher combined PEDOT:PSS/uEG loadings (PVA 15% PEDOT:PSS 15% uEG), the crystallinity and mechanical properties both decrease, reflecting the competing effects of enhanced ordering from PEDOT:PSS and disruption from uEG agglomeration, similar to the literature where increasing nanoparticle content beyond an optimal point reduces crystallinity and mechanical performance due to interfacial defects [49].

For the PVB-based yarns, the neat polymer shows a crystallinity of 34.4%, decreasing slightly to 33.5%, 32.3% and 29.1% for 10, 20 and 30% micrographite, respectively. This gradual reduction indicates that increasing mGraph loading progressively interferes with crystal growth and maintains a more disordered semicrystalline morphology, in agreement with reports on PVB/graphite or PVB/graphene composites, where plate-like carbon fillers perturb the packing of PVB chains and reduce crystallinity at higher loadings [54]. PVB yarns containing uEG show a slightly different behaviour: PVB 10% uEG retains a crystallinity comparable to neat PVB (34.3%), and PVB 20% uEG slow shows only a modest decrease to 32.9%, whereas PVB 20 uEG fast exhibits a more pronounced drop to 23.7%. The similarity between neat PVB, PVB 10% uEG and PVB 20% uEG slow suggests that uEG at moderate loadings has a limited impact on crystallinity when processed under slower take-up conditions, while the lower crystallinity for PVB 20% uEG fast likely reflects altered crystallisation kinetics under faster winding, which results in reduced time for crystal growth and increased quenching during AC electrospinning. The mechanical data for the PVB series support the view that changes in crystallinity alone cannot explain the observed strength losses. Neat PVB yarns exhibit the highest breaking stress (2798 MPa) and modulus (15.6 GPa), but both uEG- and mGraph-filled PVB yarns show large reductions in breaking stress (down to 552–1786 MPa) and normalised strength, even though crystallinity decreases by only ~5–10 percentage points. This behaviour is consistent with the glassy nature of PVB and the presence of micron-scale fillers acting as stress concentrators: the flakes disrupt the nanofibre network and create crack-initiating sites, which dominate the tensile response, while the modest reduction in crystallinity further lowers stiffness and strength. Similar behaviour has been reported in graphite- or graphene-filled glassy polymers, where conductivity and stiffness gains at high filler content come at the cost of tensile strength and ductility due to interfacial debonding and brittle crack propagation [54].

### 3.7. Cytotoxicity Test

**Figure 8:**
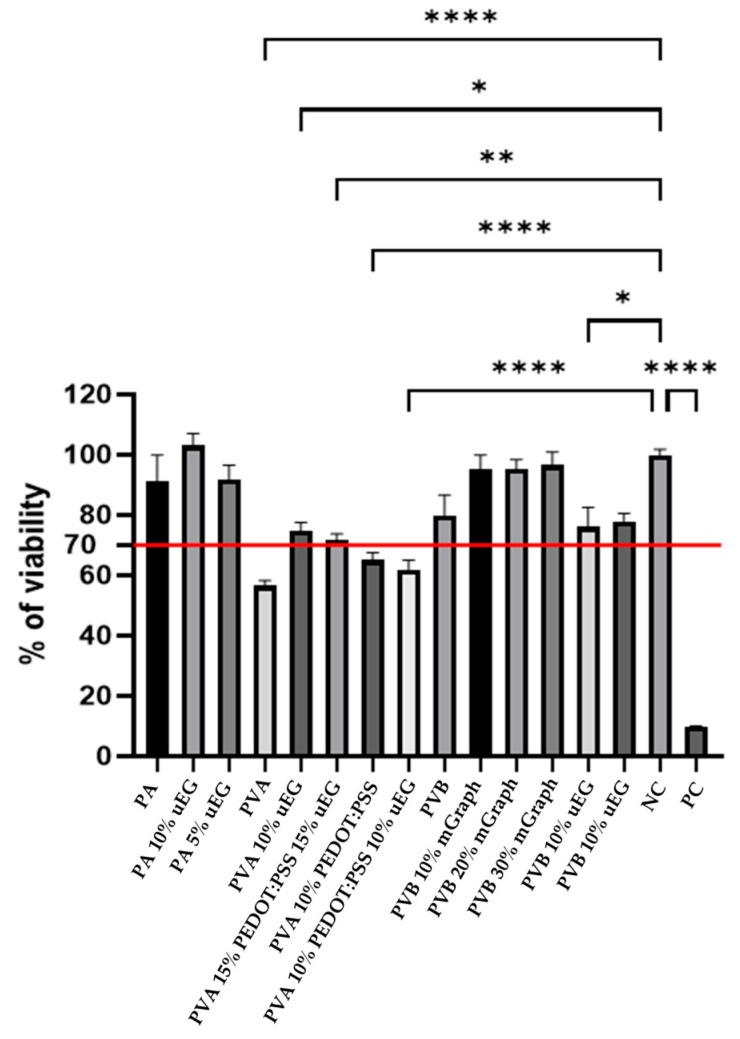
Cytotoxicity analysis of nanofibrous yarns; *, ** and **** indicates degree of difference from the control. DMEM (negative control), Triton (positive control). Data presented as mean ± SD (*n* = 8).

Cytocompatibility was assessed using a CCK-8 metabolic activity assay on extracts prepared in Dulbecco’s Modified Eagle Medium (DMEM), which served as the cell-compatible negative control, and Triton X-100, a membrane-disruptive surfactant, as the positive cytotoxicity control. DMEM represents a standard nutrient medium that maintains high viability in untreated cultures, whereas Triton X-100 induces rapid cell lysis, thereby defining the lower bound of cell survival. Following ISO- and literature-based practice, cell proliferation ≥ 70% relative to the DMEM control is interpreted as non-cytotoxic, allowing us to benchmark the nanofibrous yarn extracts against established biocompatibility criteria. One-way ANOVA testing was performed on the samples to determine differences between sample groups; the significance is indicated in the upper part of Figure 8. For this test, the PVA samples were not crosslinked, which caused them to degrade in aqueous DMEM and resulted in the expected cytotoxic effects [22].

One-way ANOVA on the absorbance values confirmed significant differences in cell viability between the sample groups (F = 235.54, *p* < 0.0001), with most formulations differing significantly from both the DMEM negative control and Triton positive control (*p* < 0.05). Overall, the addition of graphite reduced cytotoxicity, with the highest uEG loading and micrographite consistently delivering the lowest cytotoxicity, and several PVB-based graphite composites (for example, PVB 20% mGraph and PVB 30% mGraph) exhibited viability statistically comparable to the DMEM control but significantly higher than Triton (*p* < 0.01). The inclusion of PEDOT:PSS showed a similar effect, but to a lesser degree than the carbon addition, as reflected in intermediate viability values and statistically significant improvements over Triton, while remaining below the DMEM control (*p* < 0.05). PVA was intentionally used in its non-crosslinked form to study how uEG and PEDOT:PSS affect biocompatibility. The fibres swell and partially dissolve in aqueous DMEM, releasing low-molecular-weight degradation products into the medium. Pathan et al. showed that non-crosslinked electrospun PVA solutions become cytotoxic above a threshold concentration due to an increased content of low-molecular-weight fractions, whereas solutions of the original, non-electrospun PVA remained non-toxic even at high concentrations. The reduced viability observed for neat PVA extracts in our study is therefore attributed to electrospinning-induced degradation and high extractable content, rather than intrinsic toxicity of the PVA backbone, and is mitigated when graphite and PEDOT:PSS are incorporated, which stabilise the fibre morphology and reduce the amount of freely dissolving polymer [55,56,57]. This was demonstrated by samples vz 5 and 6 (PVA-based formulations with higher uEG content), which were considered non-cytotoxic as their viability was not significantly different from the DMEM control (above the 70% cell proliferation benchmark) while remaining significantly higher than the Triton control (*p* < 0.01). ANOVA between sample groups thus supports these observations, indicating that the most pronounced positive factor was uEG loading, followed by PEDOT:PSS content, in reducing cytotoxicity and restoring cell viability toward control levels.

### 3.8. Yarn Swelling Test

**Figure 9:**
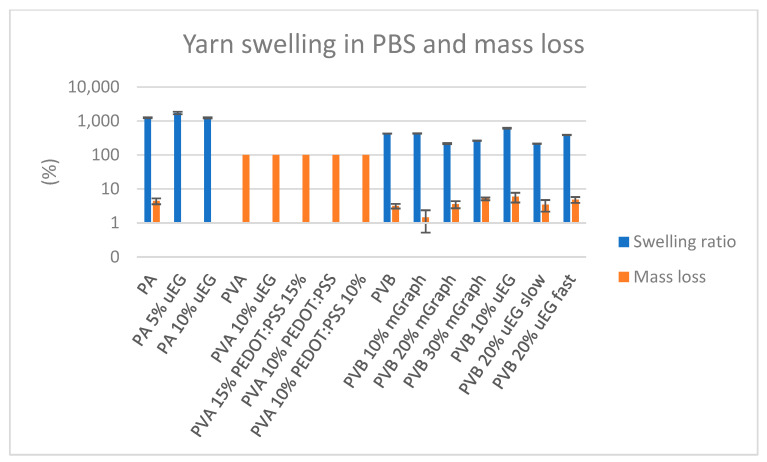
Swelling ratio and mass loss of nanofibrous yarns after 7 days in PBS (pH 7.4, 37 °C). Data presented as mean ± SD (*n* = 3).

After 7 days of immersion in PBS, all crosslinked nanofibrous yarns (PA and PVB-based) exhibited pronounced swelling while maintaining relatively low mass loss as illustrated in Figure 9. The PA yarns showed the highest swelling ratios, on the order of 1200–1700%, with the maximum value observed for the formulation containing 5% uEG. In contrast, PVB-based yarns displayed more moderate PBS uptake, with swelling generally between ~200 and 600%. Within this group, the PVB 10% uEG yarn showed the highest swelling (~612%), whereas PVB 20% mGraphite and PVB 20% uEG slow exhibited the lowest values (~215% and ~213%, respectively), suggesting that higher micrographite contents and slower production conditions result in reduced PBS liquid uptake relative to the PVB control.

Mass loss after 7 days was limited for all PA and PVB yarns, ranging from approximately 0 to 5.9%, indicating that these materials remained structurally stable in PBS over the investigated period. The largest measurable mass loss was again observed for the PVB 10% uEG yarn, while PA and PA uEG samples showed essentially no detectable degradation. By contrast, the non-crosslinked PVA-based yarns did not reach a defined swollen equilibrium state; they progressively dissolved in PBS and exhibited complete (≈100%) mass loss over 7 days, consistent with the known water solubility of uncrosslinked PVA under physiological-like conditions. Taken together, these results demonstrate that the PA 4,6 and PVB composite yarns can absorb substantial amounts of PBS while retaining their dry mass and integrity over at least one week, whereas PVA-based yarns would require appropriate crosslinking to achieve comparable aqueous stability.

## 4. Conclusions

This study has demonstrated the scalable production of nanofibrous biocompatible composite yarn systems with excellent filler retention during electrospinning. Electrical conductivity and mechanical strength underperformed due to the incorporation of microfillers into the nanofibrous system, as ultrasonication of EG did not reduce the in-plane particle size to the nanometre range. XRD confirmed that the addition of carbon fillers resulted in a reduction in matrix crystallinity; however, this alone was not responsible for the observed strength reduction. The addition of PEDOT:PSS, however, resulted in a more crystalline structure, enhancing the mechanical strength of the nanofibrous yarn. uEG exceeded micrographite in decreasing resistivity within electrospun yarns, corroborating the assertion that optimised dispersion and nanoscale network formation are essential for achieving high conductivity. Further processing of EG to achieve nanoscale dimensions, such as via ball milling, is expected to improve this by better incorporation into the nanofibers. The combination with PEDOT:PSS further enhances performance, although it remains inferior to conductivities observed in nanocarbon or metallic fibre systems. Although it has lower electrical performance, it is more economical to produce these nanocarbon structures and boasts better biocompatibility compared to most conductive metallic particle systems. The addition of uEG, micrographite, and PEDOT:PSS reduced the material’s cytotoxicity by promoting cell proliferation, even outperforming non-stabilised PVA, demonstrating the material’s excellent biocompatibility, therefore making it very suitable for biomedical applications.

## Figures and Tables

**Figure 4:**
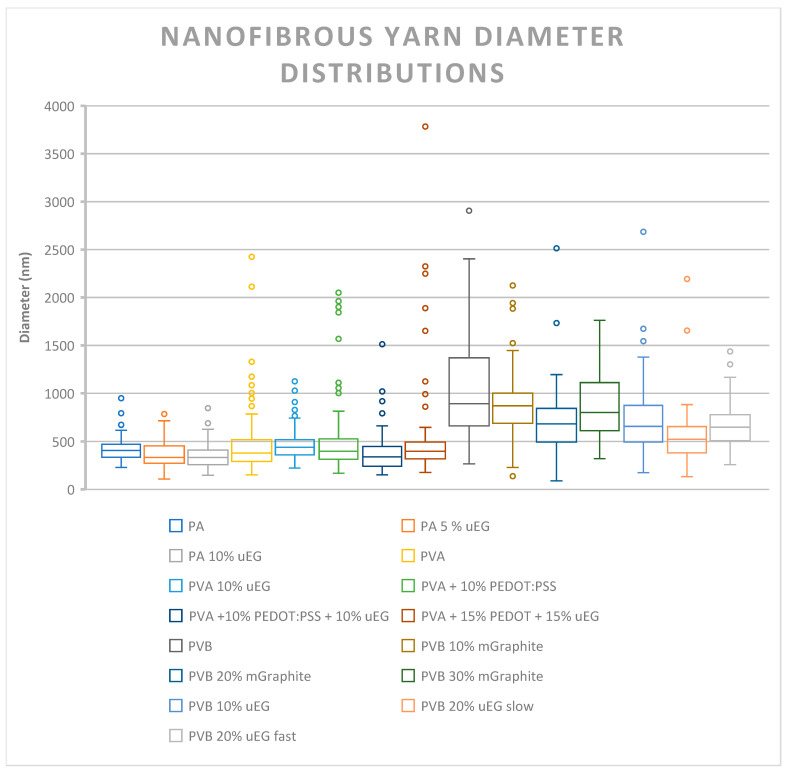
Box plots of yarn diameter distributions (nm) for nanofibrous composite samples (*n* = 100 measurements per sample).

**Table 1:** TGA results of nanofibrous yarns. * For “PVA 15% PEDOT:PSS 15% uEG”, the uEG value is calculated with the residue value subtracted from “PVA 10% PEDOT:PSS”.

Sample	%Residue	%uEG/mGraph in Sample
PA	1.57	0.00
PA 5% uEG	3.56	1.99
PA 10% uEG	9.90	8.33
PVB	3.28	0.00
PVB 10% mGraph	10.16	6.88
PVB 20% mGraph	21.19	17.91
PVB 30% mGraph	32.94	29.66
PVB 10% uEG	10.10	6.81
PVB 20% uEG fast	12.97	9.69
PVB 20% uEG slow	10.66	7.38
PVA	5.11	0.00
PVA 10% uEG	10.31	5.20
PVA 10% PEDOT:PSS	2.98	0.00
PVA 10% PEDOT:PSS 10% uEG	7.199	4.22
PVA 15% PEDOT:PSS 15% uEG	11.50	8.52 *

**Table 2:** Electrical measurement data, cross-sectional area of nanofibrous yarns and linear weight (Tex).

Sample	R of 48 Parallel Yarns (Ω)	CSA of a Single Yarn (µm^2^)	R of Single Yarn (Ω)	ρ (Ω·m)	σ (S/m)	Tex (g/1000 m)	ρ/Tex (Ω·m/Tex)
PA	1.78 × 10^12^	639.6	8.54 × 10^13^	5.46 × 10^6^	1.83 × 10^−7^	39.58	2.16 × 10^8^
PA 5 uEG	2.84 × 10^11^	293	1.36 × 10^13^	3.99 × 10^5^	2.51 × 10^−6^	26.04	1.04 × 10^7^
PA 10 uEG	4.01 × 10^10^	515.4	1.92 × 10^12^	9.92 × 10^4^	1.01 × 10^−5^	11.84	1.17 × 10^6^
PVB	1.71 × 10^12^	439.5	8.21 × 10^13^	3.61 × 10^6^	2.77 × 10^−7^	63.68	2.30 × 10^8^
PVB 10 mGraph	8.56 × 10^11^	448.1	4.11 × 10^13^	1.84 × 10^6^	5.43 × 10^−7^	59.34	1.09 × 10^8^
PVB 20 mGraph	1.31 × 10^10^	707.8	6.29 × 10^11^	4.45 × 10^4^	2.25 × 10^−5^	78.76	3.51 × 10^6^
PVB 30 mGraph	3.80 × 10^9^	1140.5	1.82 × 10^11^	2.08 × 10^4^	4.81 × 10^−5^	66.02	1.37 × 10^6^
PVB 10 uEG	6.61 × 10^10^	356.1	3.17 × 10^12^	1.13 × 10^5^	8.85 × 10^−6^	42.4	4.79 × 10^6^
PVB 20 uEG fast	7.30 × 10^9^	357.5	3.50 × 10^11^	1.25 × 10^4^	8.00 × 10^−5^	41.84	5.24 × 10^5^
PVB 20 uEG slow	1.94 × 10^10^	357.5	9.31 × 10^11^	3.33 × 10^4^	3.00 × 10^−5^	19.52	6.50 × 10^5^
PVA	3.61 × 10^11^	452.4	1.73 × 10^13^	7.84 × 10^5^	1.28 × 10^−6^	46.62	3.65 × 10^7^
PVA 10 uEG	3.38 × 10^10^	351.5	1.62 × 10^12^	5.70 × 10^4^	1.75 × 10^−5^	24.54	1.40 × 10^6^
PVA 10 PEDOT:PSS	5.84 × 10^9^	600.5	2.80 × 10^11^	1.68 × 10^4^	5.95 × 10^−5^	36.3	6.11 × 10^5^
PVA 10 PEDOT:PSS 10 uEG	2.88 × 10^10^	372.7	1.38 × 10^12^	5.15 × 10^4^	1.94 × 10^−5^	17.84	9.19 × 10^5^
PVA 15 PEDOT:PSS 15 uEG	4.58 × 10^9^	429.3	2.20 × 10^11^	9.44 × 10^3^	1.06 × 10^−4^	14.5	1.37 × 10^5^

**Table 3:** Tensile measurements of samples with standard deviations indicated in brackets.

Sample	Measured Breaking Force (N)	Breaking Stress, σ (MPa)	σ/Tex (MPa/Tex)	Modulus, E (MPa)	E/Tex (MPa/Tex)	Elong. (%)
PA	1.08 (0.16)	1689 (250)	43 (6)	13,714 (2002)	346 (51)	29.6 (3.1)
PA 5 uEG	0.68 (0.07)	2321 (239)	89 (9)	8616 (939)	331 (36)	26.4 (2.6)
PA 10 uEG	0.18 (0.03)	349 (58)	29 (5)	2296 (358)	194 (30)	16.5 (4.2)
PVA	0.73 (0.07)	1614 (155)	35 (3)	9248 (858)	198 (18)	14.6 (2.3)
PVA 10 uEG	0.43 (0.08)	1223 (228)	50 (9)	5518 (1051)	225 (43)	64.2 (6.4)
PVA 10 PEDOTPSS	1.22 (0.13)	2031 (216)	56 (6)	15,508 (1594)	427 (44)	30.8 (5.2)
PVA 10 PEDOTPSS 10 uEG	0.62 (0.04)	1664 (107)	93 (6)	7951 (546)	446 (31)	13.0 (6.0)
PVA 15 PEDOTPSS 15 uEG	0.42 (0.05)	978 (116)	67 (8)	5363 (659)	370 (45)	21.4 (2.6)
PVB	1.23 (0.16)	2798 (364)	44 (6)	15,613 (2070)	245 (33)	53.5 (11.6)
PVB 10 uEG	0.29 (0.05)	814 (140)	19 (3)	3678 (700)	87 (17)	28.7 (6.1)
PVB 20 uEG slow	0.30 (0.03)	839 (84)	43 (4)	3780 (393)	194 (20)	25.3 (6.0)
PVB 20 uEG fast	0.31 (0.06)	867 (168)	21 (4)	3965 (738)	95 (18)	31.3 (8.3)
PVB 10 mGraph	0.80 (0.17)	1786 (379)	30 (6)	10,186 (2216)	172 (37)	33.7 (9.0)
PVB 20 mGraph	1.02 (0.17)	1441 (240)	18 (3)	13,007 (2167)	165 (28)	25.1 (6.1)
PVB 30 mGraph	0.63 (0.12)	552 (105)	8 (2)	8059 (1479)	122 (22)	27.4 (9.5)

## Data Availability

All data generated are presented in the manuscript and Supplementary File.

## Supplementary Materials

The following supporting information can be downloaded at https://www.mdpi.com/article/10.3390/polym18101225/s1. Table S1: Nanofiber yarn diameter analysis summary.; Figure S1: Yarn diameter histogram of nanofiber yarn sample: PA; Figure S2: Yarn diameter histogram of nanofiber yarn sample: PA 10% ultrasonicated EG; Figure S3: Yarn diameter histogram of nanofiber yarn sample: PA 5% ultrasonicated EG; Figure S4: Yarn diameter histogram of nanofiber yarn sample: PVA; Figure S5: Yarn diameter histogram of nanofiber yarn sample: PVA 10% ultrasonicated EG; Figure S6: Yarn diameter histogram of nanofiber yarn sample: PVA 15% PEDOT:PSS 15% ultrasonicated EG; Figure S7: Yarn diameter histogram of nanofiber yarn sample: PVA 10% PEDOT:PSS; Figure S8: Yarn diameter histogram of nanofiber yarn sample: PVA 10% PEDOT:PSS 10% ultrasonicated EG; Figure S9: Yarn diameter histogram of nanofiber yarn sample: PVB; Figure S10: Yarn diameter histogram of nanofiber yarn sample: PVB 10% micrographite; Figure S11: Yarn diameter histogram of nanofiber yarn sample: PVB 20% micrographite; Figure S12: Yarn diameter histogram of nanofiber yarn sample: PVB 30% micrographite; Figure S13: Yarn diameter histogram of nanofiber yarn sample: PVB 10% ultrasonicated EG; Figure S14: Yarn diameter histogram of nanofiber yarn sample: PVB 20% ultrasonicated EG with slow processing speed; Figure S15: Yarn diameter histogram of nanofiber yarn sample: PVB 20% ultrasonicated EG with faster processing speed; Figure S16: TGA spectra of PA nanofibrous yarn samples; Figure S17: TGA spectra of PVA nanofibrous yarn samples; Figure S18: TGA spectra of PVB nanofibrous yarn samples; Figure S19: FTIR of PA based nanofibrous yarns; Figure S20: FTIR of PVB based nanofibrous yarns; Figure S21: FTIR of PVA based nanofibrous yarns; Figure S22: X-Ray Diffraction spectra of PA nanofibrous yarn sample; Figure S23: Diffraction spectra of PA 5% uEG nanofibrous yarn sample; Figure S24: X-Ray Diffraction spectra of PA 10% uEG nanofibrous yarn sample; Figure S25: X-Ray Diffraction spectra of PVA nanofibrous yarn sample; Figure S26: X-Ray Diffraction spectra of PVA 10% uEG nanofibrous yarn sample; Figure S27: X-Ray Diffraction spectra of PVA 10% PEDOT:PSS nanofibrous yarn sample; Figure S28: X-Ray Diffraction spectra of PVA 10% PEDOT:PSS 10% uEG nanofibrous yarn sample; Figure S29: X-Ray Diffraction spectra of PVA 15% PEDOT:PSS 15% uEG nanofibrous yarn sample; Figure S30: X-Ray Diffraction spectra of PVB nanofibrous yarn sample; Figure S31: X-Ray Diffraction spectra of PVB 10% micro graphite nanofibrous yarn sample; Figure S32: X-Ray Diffraction spectra of PVB 20% micro graphite nanofibrous yarn sample; Figure S33: X-Ray Diffraction spectra of PVB 30% micro graphite nanofibrous yarn sample; Figure S34: X-Ray Diffraction spectra of PVB 10% uEG nanofibrous yarn sample; Figure S35: X-Ray Diffraction spectra of PVB 20% uEG slow nanofibrous yarn sample; Figure S36: X-Ray Diffraction spectra of PVB 20% uEG fast nanofibrous yarn sample.

## Abbreviations

The following abbreviations are used in this manuscript:

**Abbreviation**	**Full Form/Description**
AC	Alternating Current
ANOVA	Analysis of Variance
BF	Breaking Force
CSA	Cross-Sectional Area
DMEM	Dulbecco’s Modified Eagle Medium
E	Modulus (Young’s modulus)
EG	Expanded Graphite
Elong.	Elongation at Break (%)
NC	Negative Control
PA	Polyamide (PA 4,6 Stanyl)
PCL	Polycaprolactone
PEDOT:PSS	poly(3,4-ethylenedioxythiophene):polystyrene sulfonate
PVA	Polyvinyl Alcohol (125k Mw, 89% dehydrolysed)
PVB	Polyvinyl Butyral (55k Mw)
R	Resistance
RH	Relative Humidity
SEM	Scanning Electron Microscopy
TGA	Thermogravimetric Analysis
TUL	Technical University of Liberec
XRD	X-ray Diffraction
mGraph	Micrographite
rpm	Revolutions per Minute
uEG	Ultrasonicated Expanded Graphite
Ïƒ	Breaking Stress/Strength (MPa)
Ïƒ/Tex	Normalized Breaking Stress (MPa/tex)
