# Supplementary material for: Scalable AC Electrospinning of Biocompatible Nanofibrous Yarns Based on Expanded Graphite and PEDOT:PSS
Source: Polymers (Basel). 2026 May 17;18(10):1225. doi: 10.3390/polym18101225 (PMC13211241; doi:10.3390/polym18101225)
Supplement: Supplementary file 1 [file polymers-18-01225-s001.zip › polymers-4252544-supplementary.pdf]

This document contains the nanofiber yarn diameter analysis summary table and histograms, TGA curves, FTIR - and XRD spectra.

Nanofiber yarn diameter analysis

Table S1: Nanofiber yarn diameter analysis summary.

Sample	Mean	Median	SD	CV %	IQR	Outliers %
PA	422.1	406.7	129	30.7	132	6
PA 5% uEG	369.4	332.3	136	36.7	177	1
PA 10% uEG	350.5	334.2	123	35.2	148	2
PVA	471	378.1	338	71.7	225	8
PVA 10% uEG	466.4	439	159	34	158	5
PVA + 10% PEDOT:PSS	508.1	398.6	367	72.2	202	9
PVA +10% PEDOT:PSS + 10% uEG	386.7	339.9	212	54.8	203	7
PVA + 15% PEDOT + 15% uEG	550.7	397.2	535	97.1	173	12
PVB	1039	891.4	526	50.7	682	3
PVB 10% mGraphite	877	870.3	356	40.6	312	10
PVB 20% mGraphite	694.8	681.9	311	44.8	336	2
PVB 30% mGraphite	874.8	800.1	316	36.2	488	0
PVB 10% uEG	717.9	655.6	356	49.6	373	3
PVB 20% uEG slow	537.5	523.7	266	49.4	260	2
PVB 20% uEG fast	665.8	648.8	216	32.5	267	2

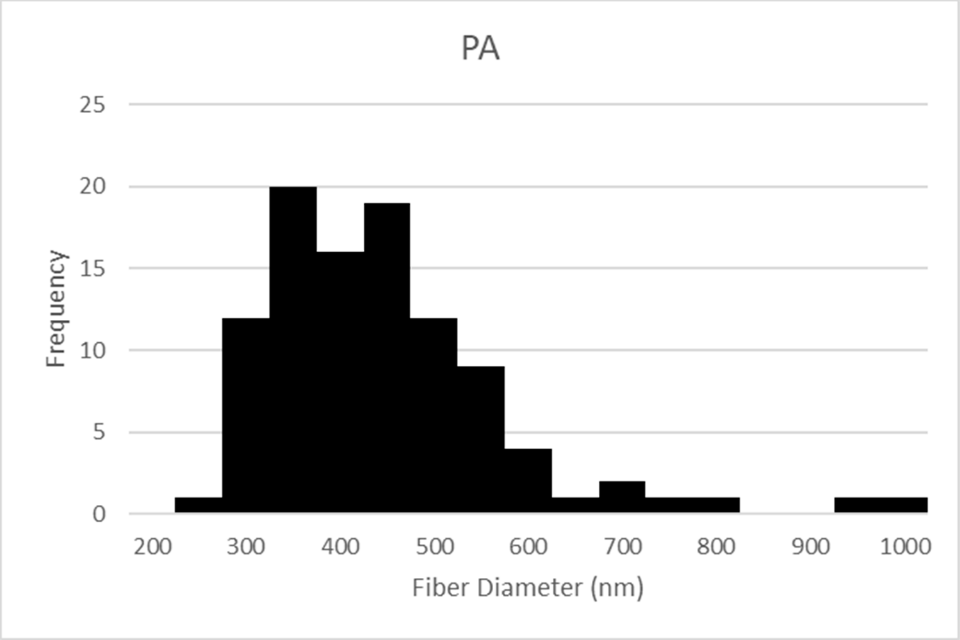

Figure S1: Yarn diameter histogram of nanofiber yarn sample: PA

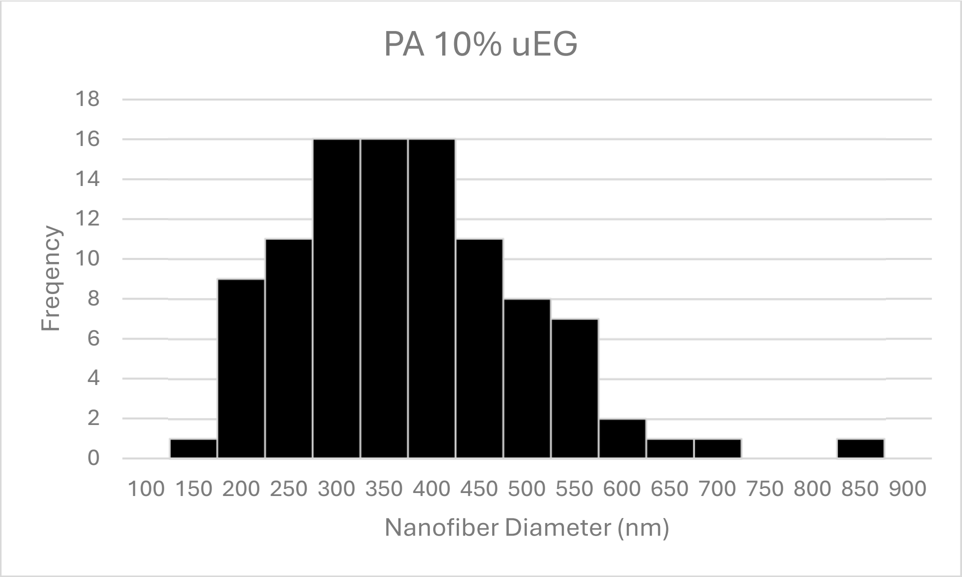

Figure S2: Yarn diameter histogram of nanofiber yarn sample: PA 10% ultrasonicated EG

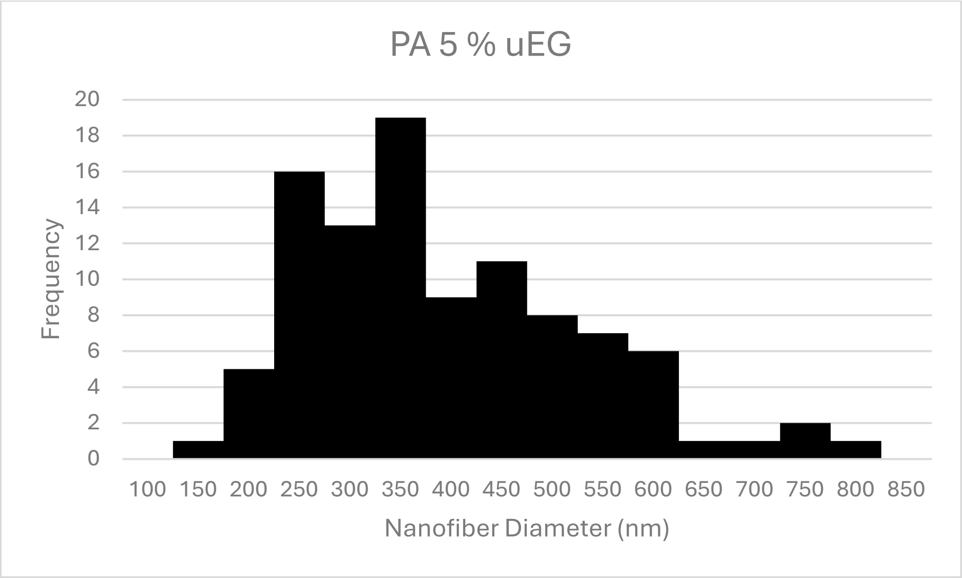

Figure S3: Yarn diameter histogram of nanofiber yarn sample: PA 5% ultrasonicated EG

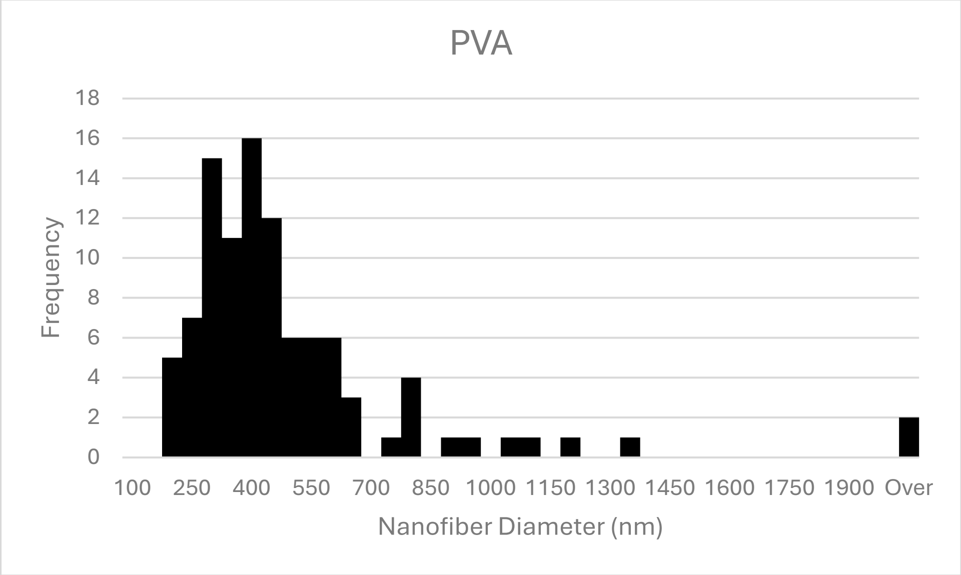

Figure S4: Yarn diameter histogram of nanofiber yarn sample: PVA

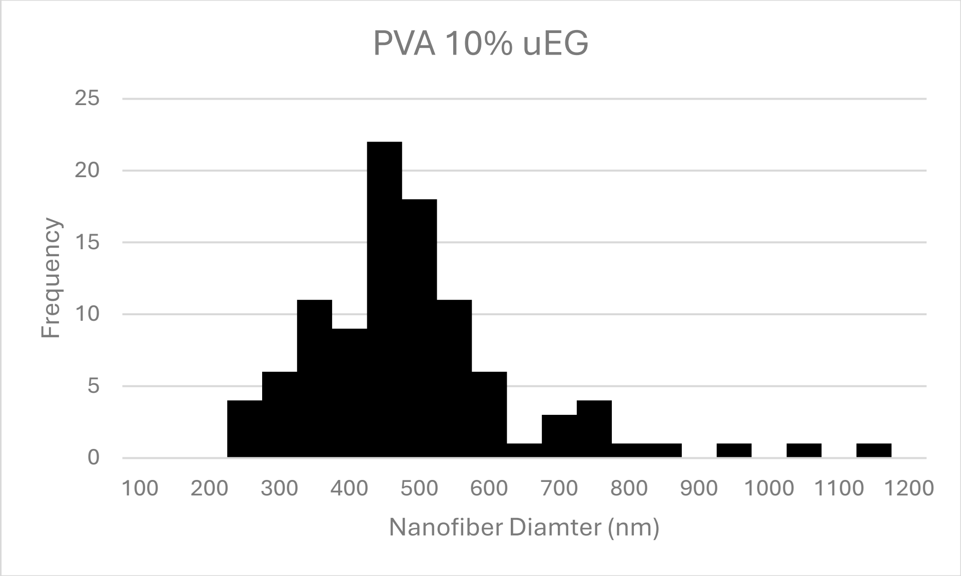

Figure S5: Yarn diameter histogram of nanofiber yarn sample: PVA 10% ultrasonicated EG

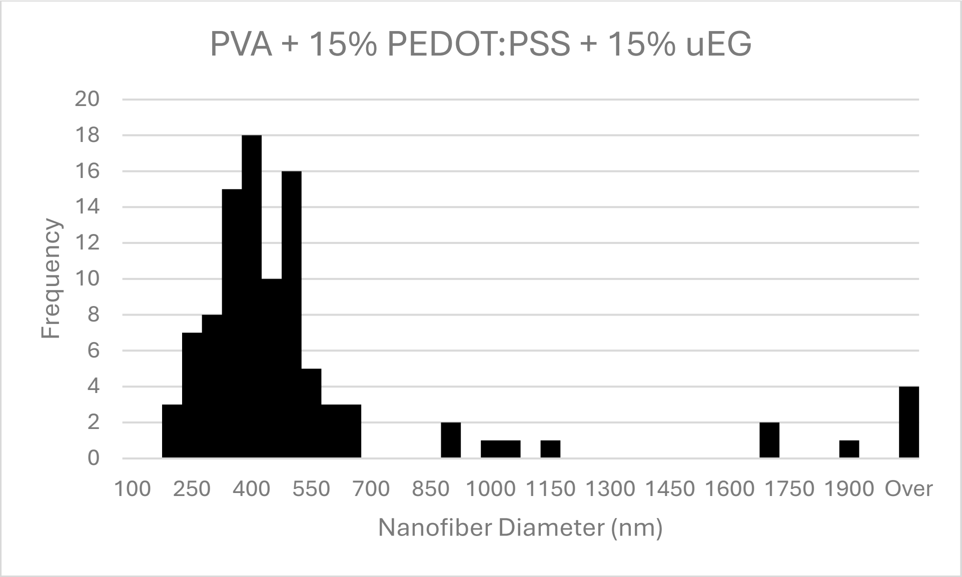

Figure S6: Yarn diameter histogram of nanofiber yarn sample: PVA 15% PEDOT:PSS 15% ultrasonicated EG

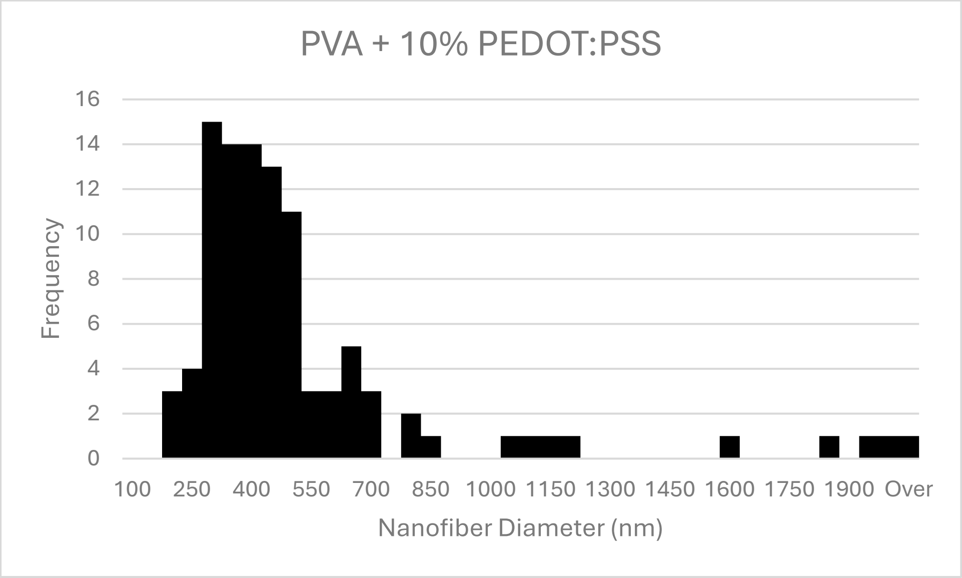

Figure S7: Yarn diameter histogram of nanofiber yarn sample: PVA 10% PEDOT:PSS

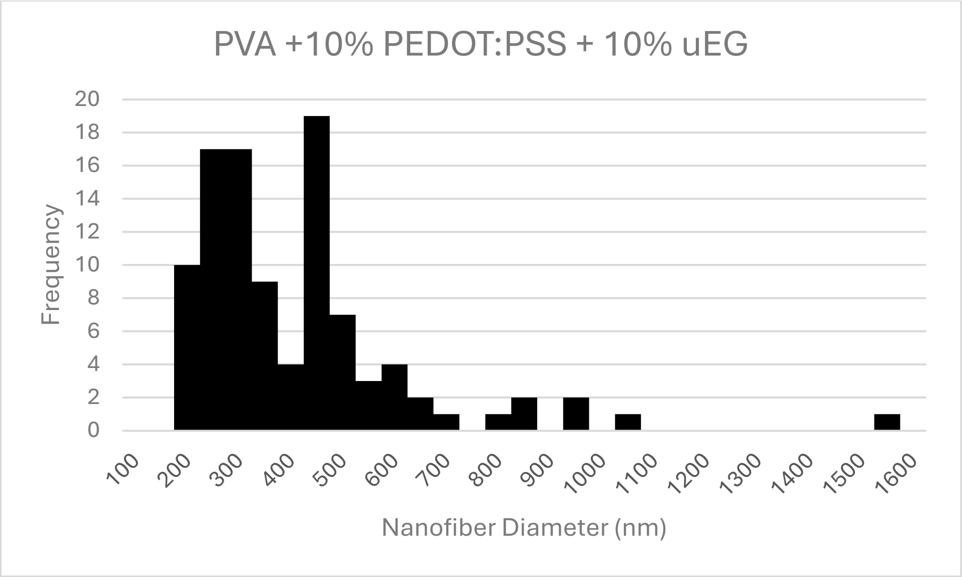

Figure S8: Yarn diameter histogram of nanofiber yarn sample: PVA 10% PEDOT:PSS 10% ultrasonicated EG

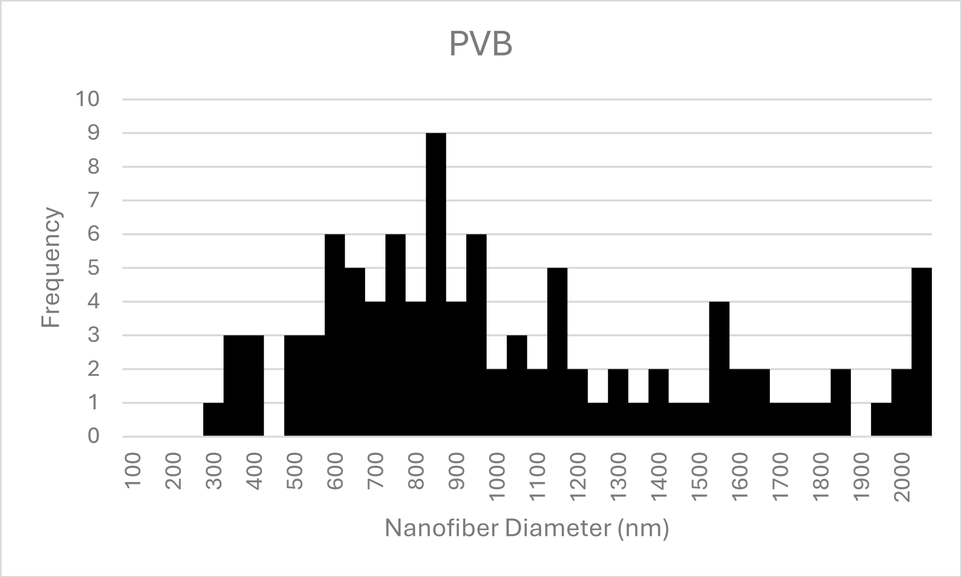

Figure S9: Yarn diameter histogram of nanofiber yarn sample: PVB

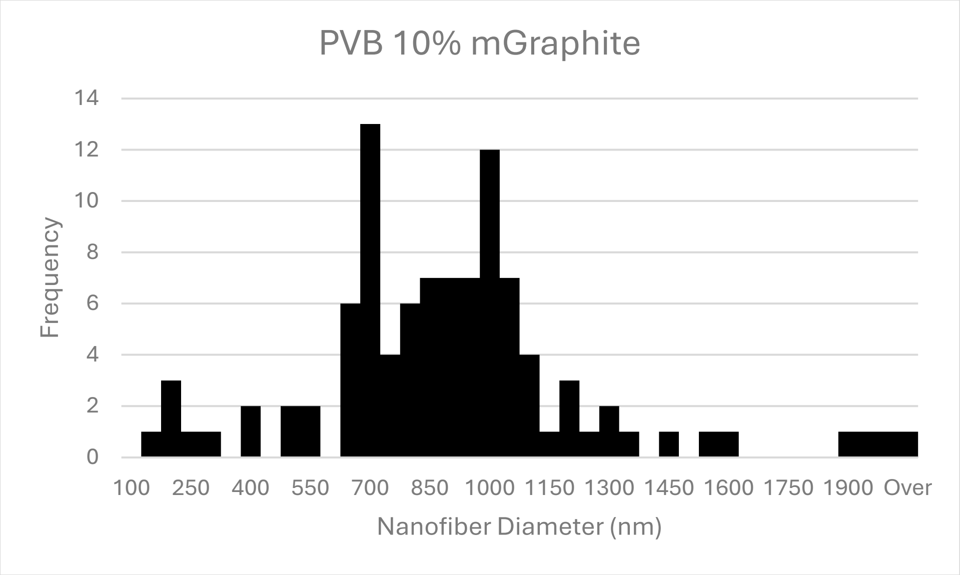

Figure S10: Yarn diameter histogram of nanofiber yarn sample: PVB 10% micrographite

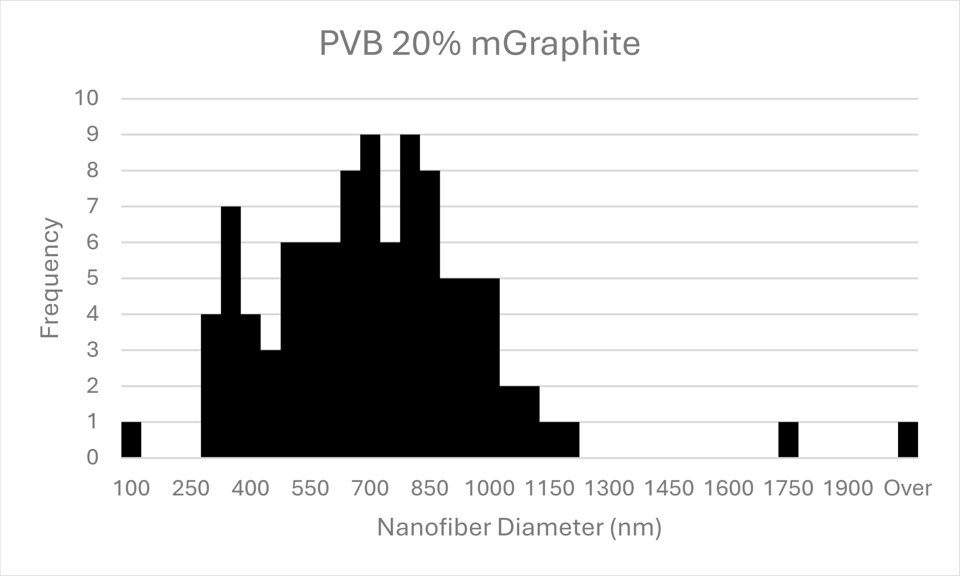

Figure S11: Yarn diameter histogram of nanofiber yarn sample: PVB 20% micrographite

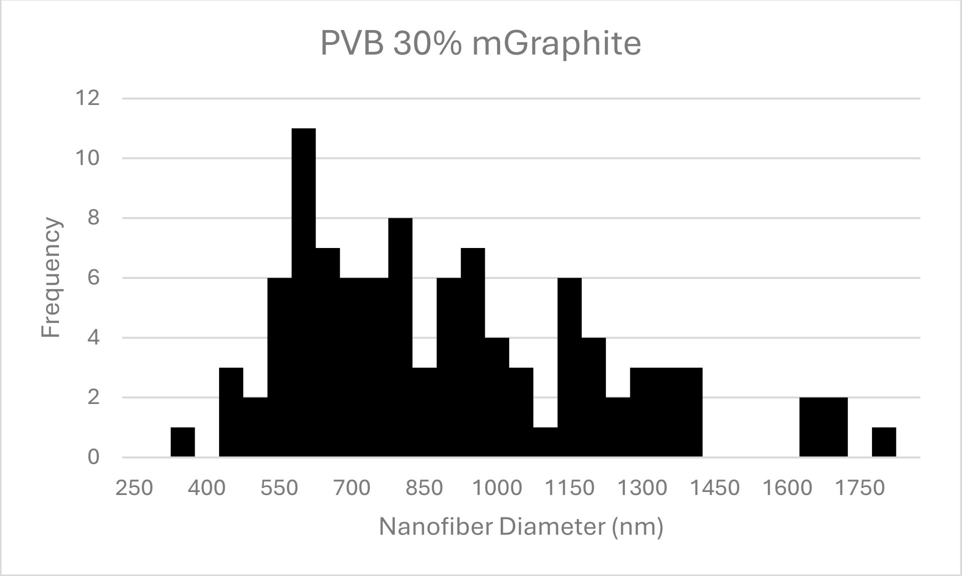

Figure S12: Yarn diameter histogram of nanofiber yarn sample: PVB 30% micrographite

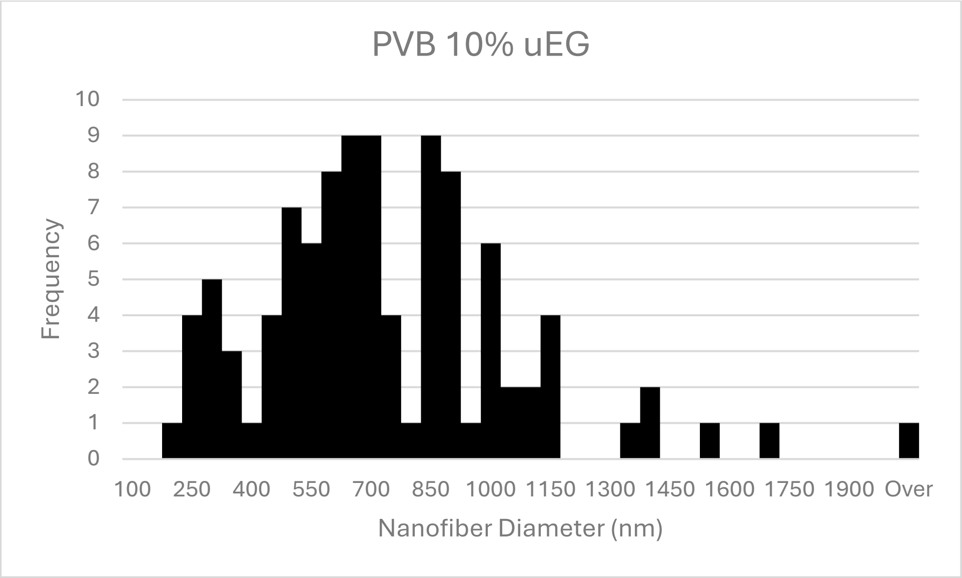

Figure S13: Yarn diameter histogram of nanofiber yarn sample: PVB 10% ultrasonicated EG

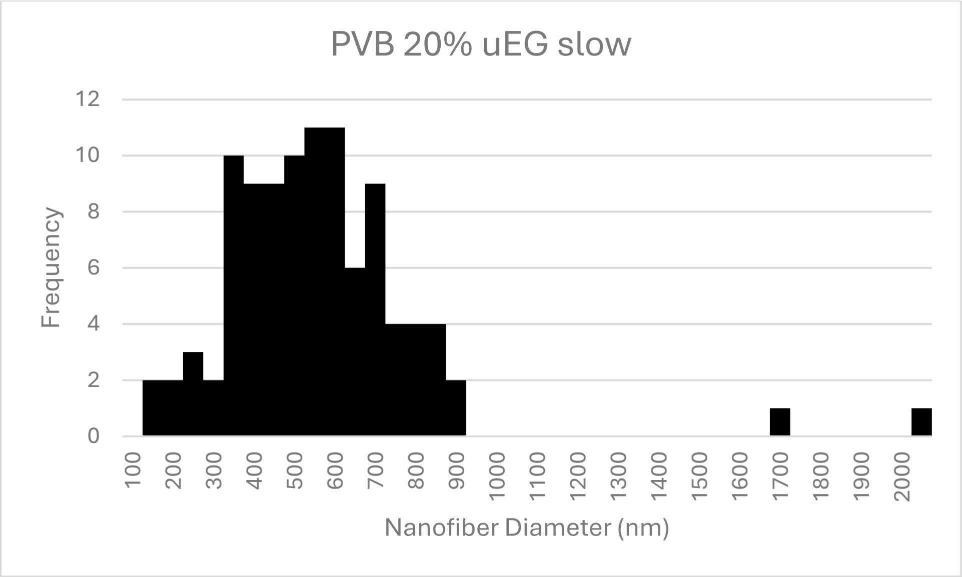

Figure S14: Yarn diameter histogram of nanofiber yarn sample: PVB 20% ultrasonicated EG with slow processing speed

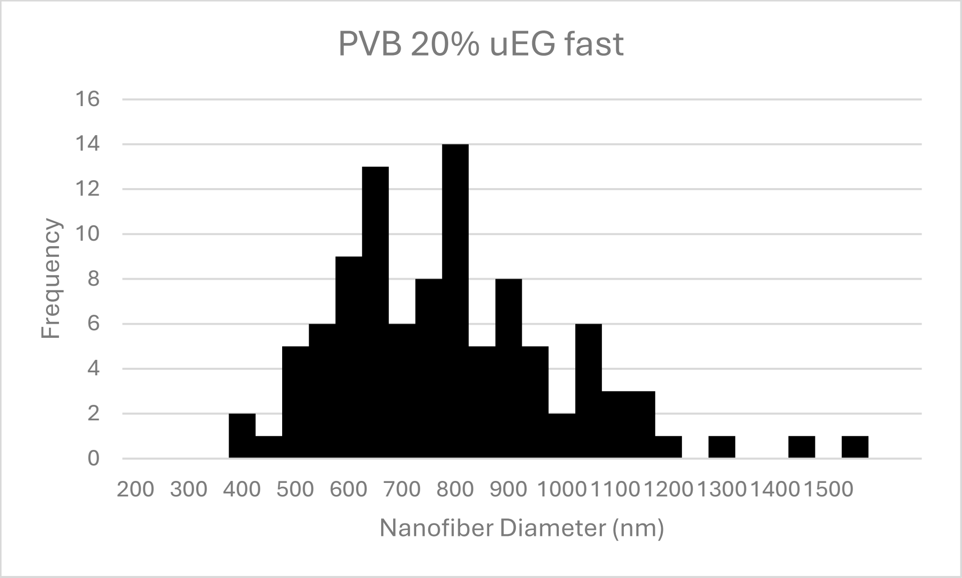

Figure S15: Yarn diameter histogram of nanofiber yarn sample: PVB 20% ultrasonicated EG with faster processing speed

TGA Curves

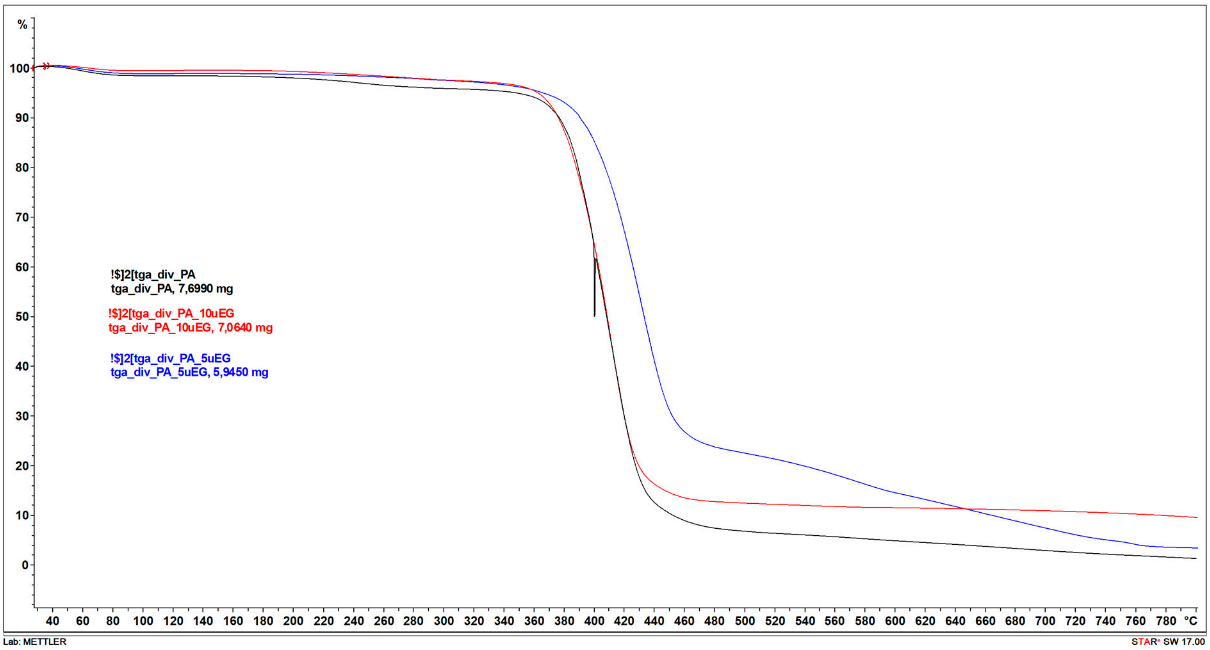

Figure S16: TGA spectra of PA nanofibrous yarn samples

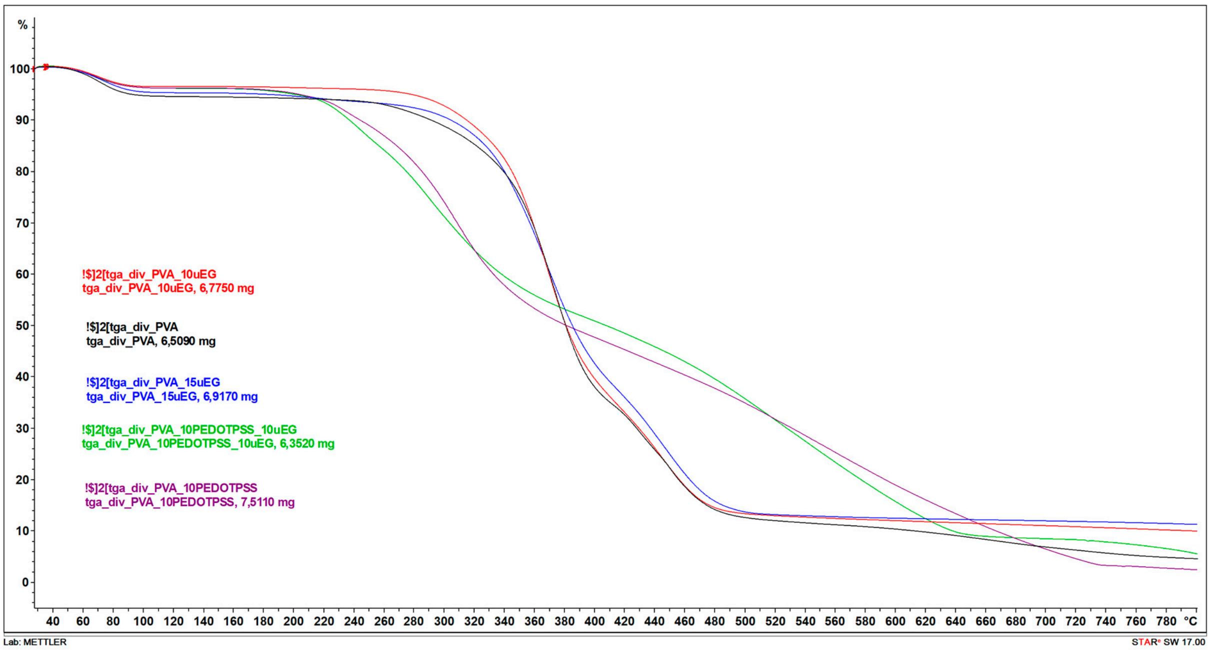

Figure S17: TGA spectra of PVA nanofibrous yarn samples

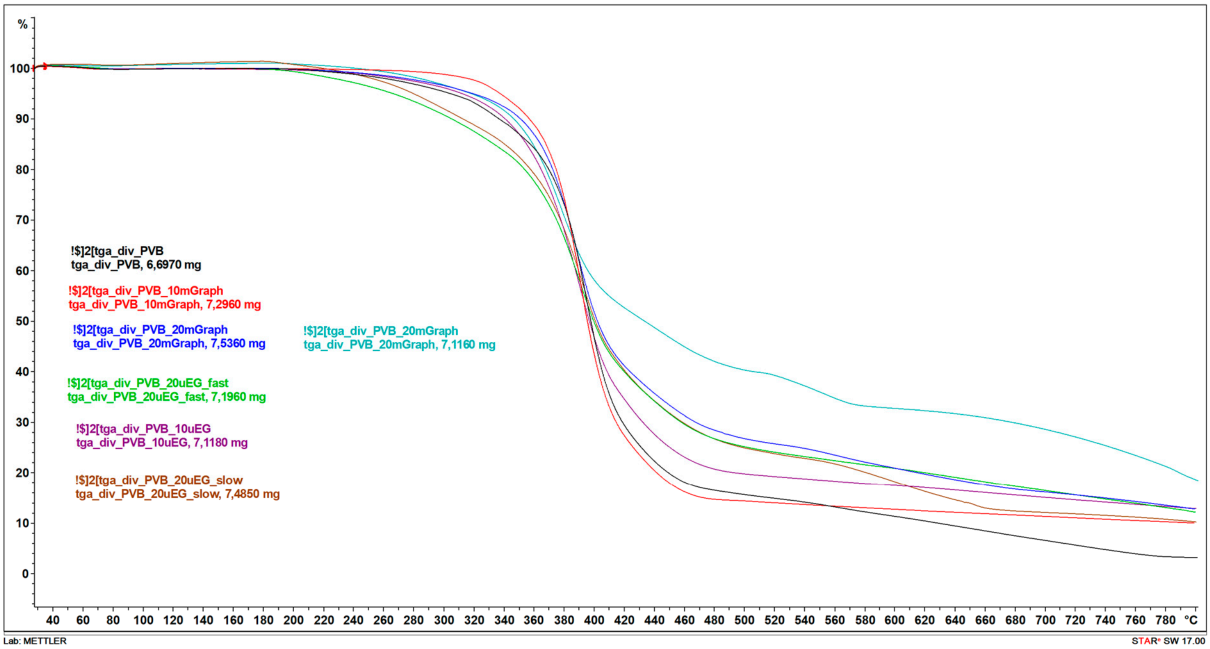

Figure S18: TGA spectra of PVB nanofibrous yarn samples

Nanofiber yarn diameter analysis

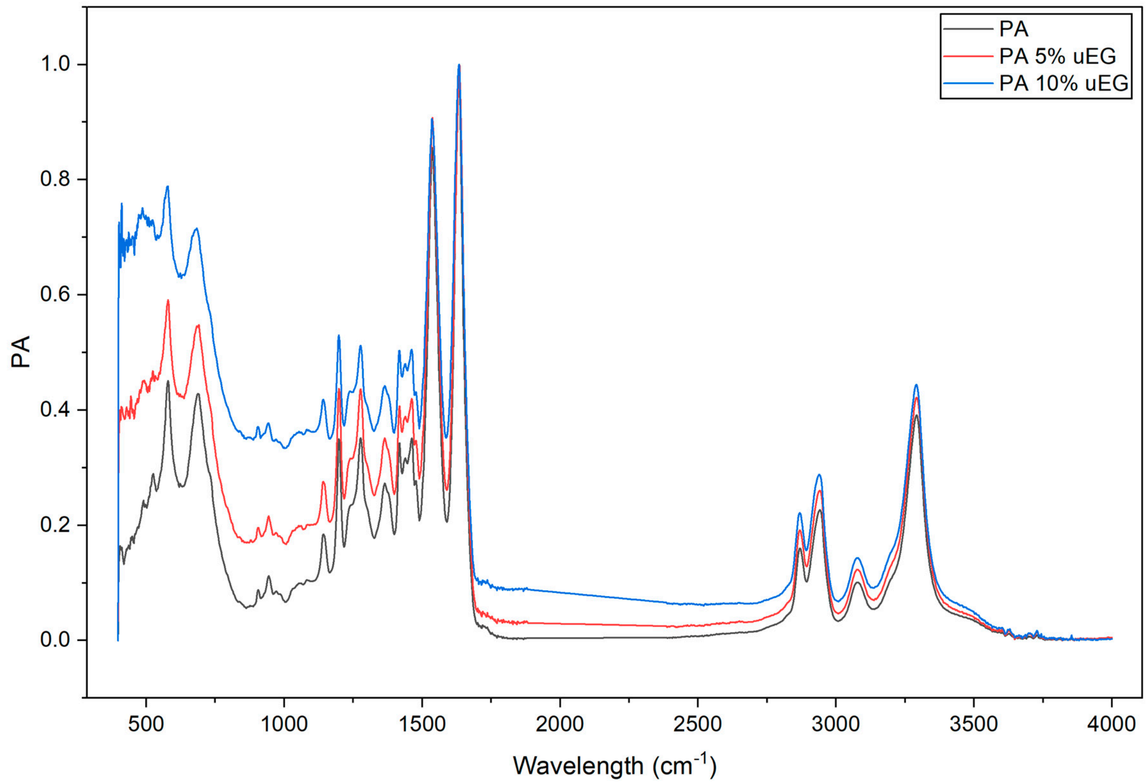

Figure S19: FTIR of PA based nanofibrous yarns

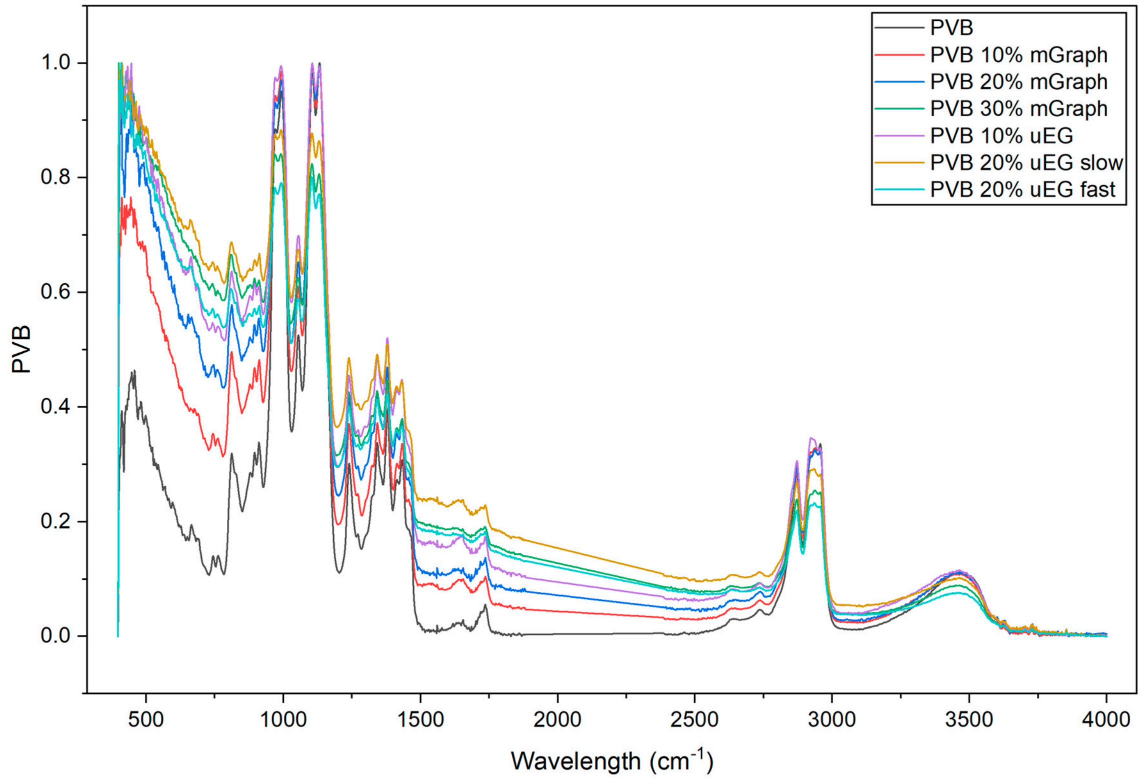

Figure S20: FTIR of PVB based nanofibrous yarns

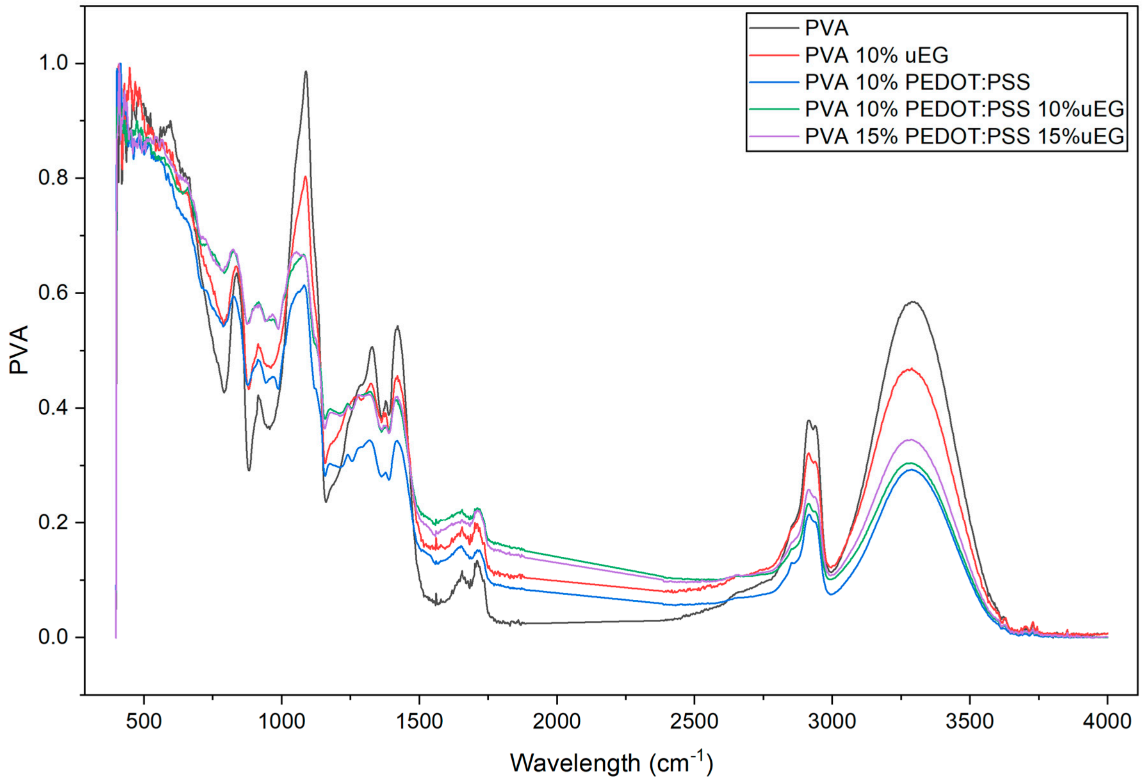

Figure S21: FTIR of PVA based nanofibrous yarns

XRD Spectra

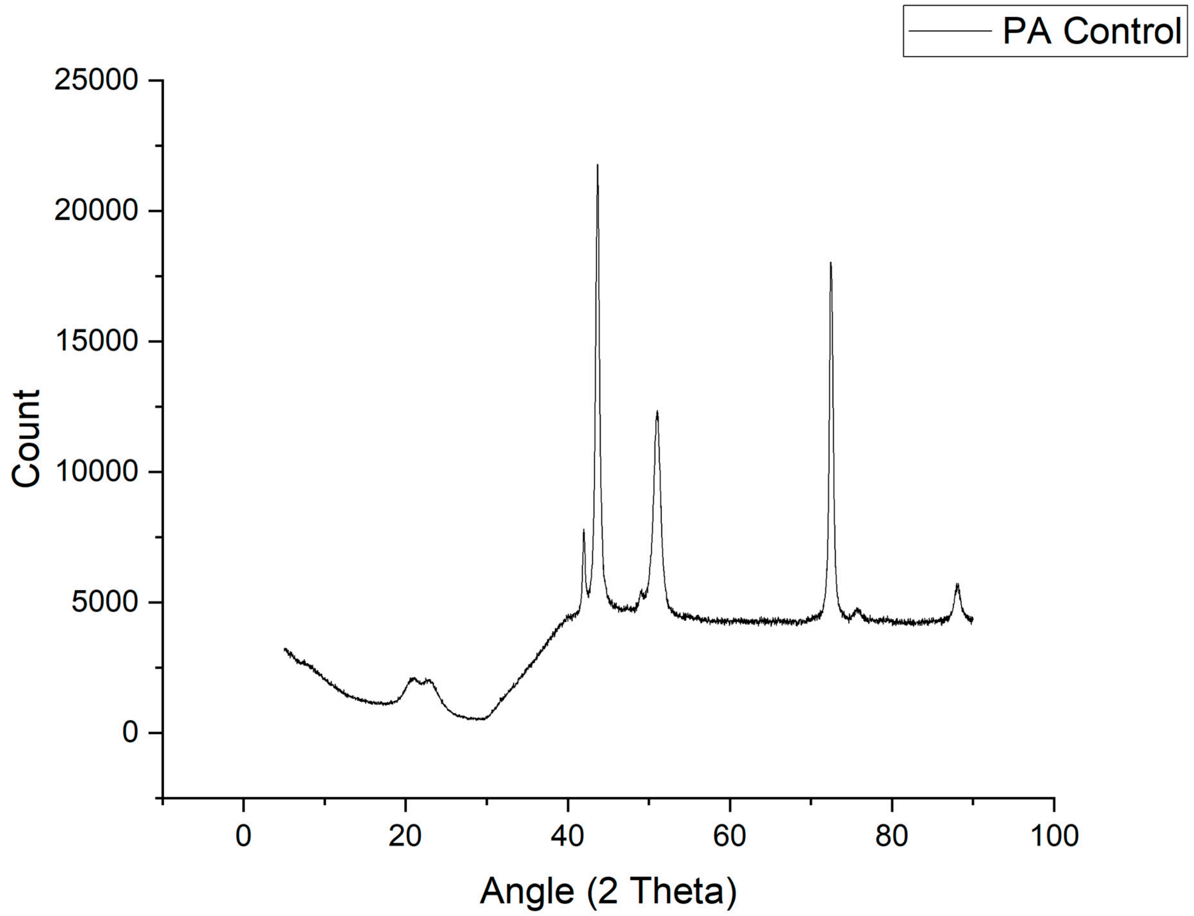

Figure S22: X-Ray Diffraction spectra of PA nanofibrous yarn sample

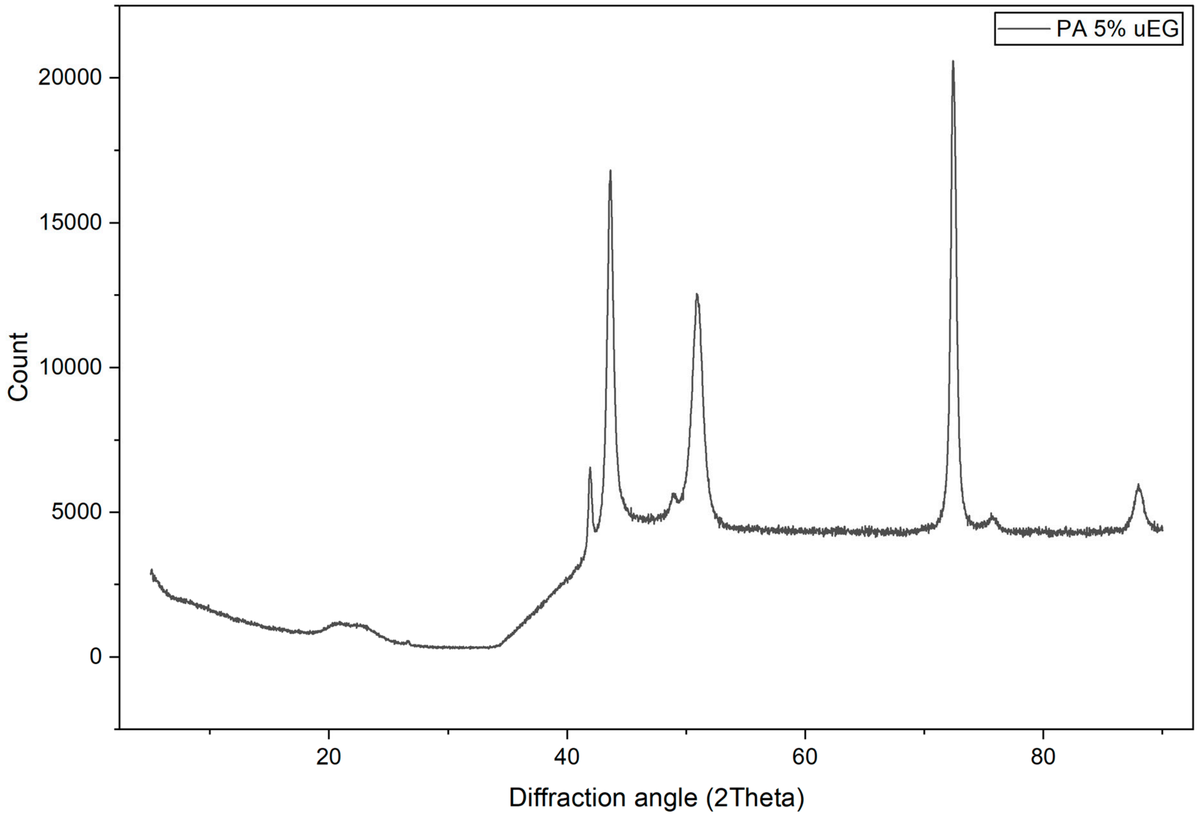

Figure S23: Diffraction spectra of PA 5% uEG nanofibrous yarn sample

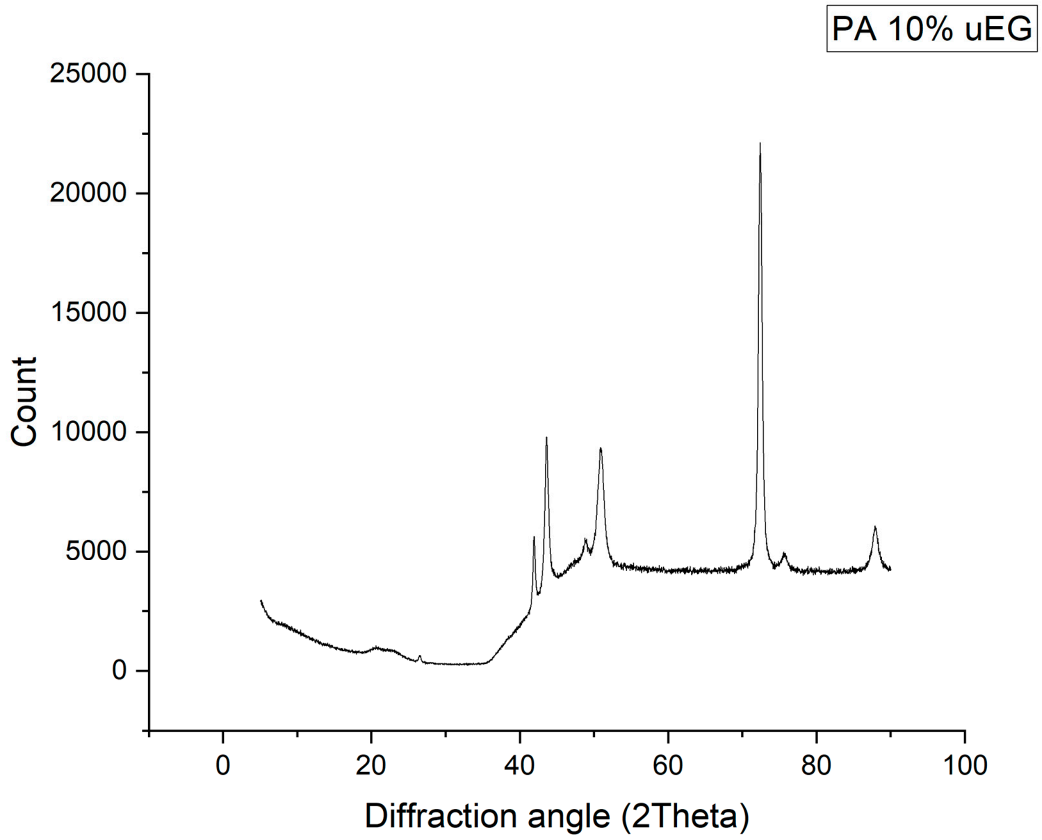

Figure S24: X-Ray Diffraction spectra of PA 10% uEG nanofibrous yarn sample

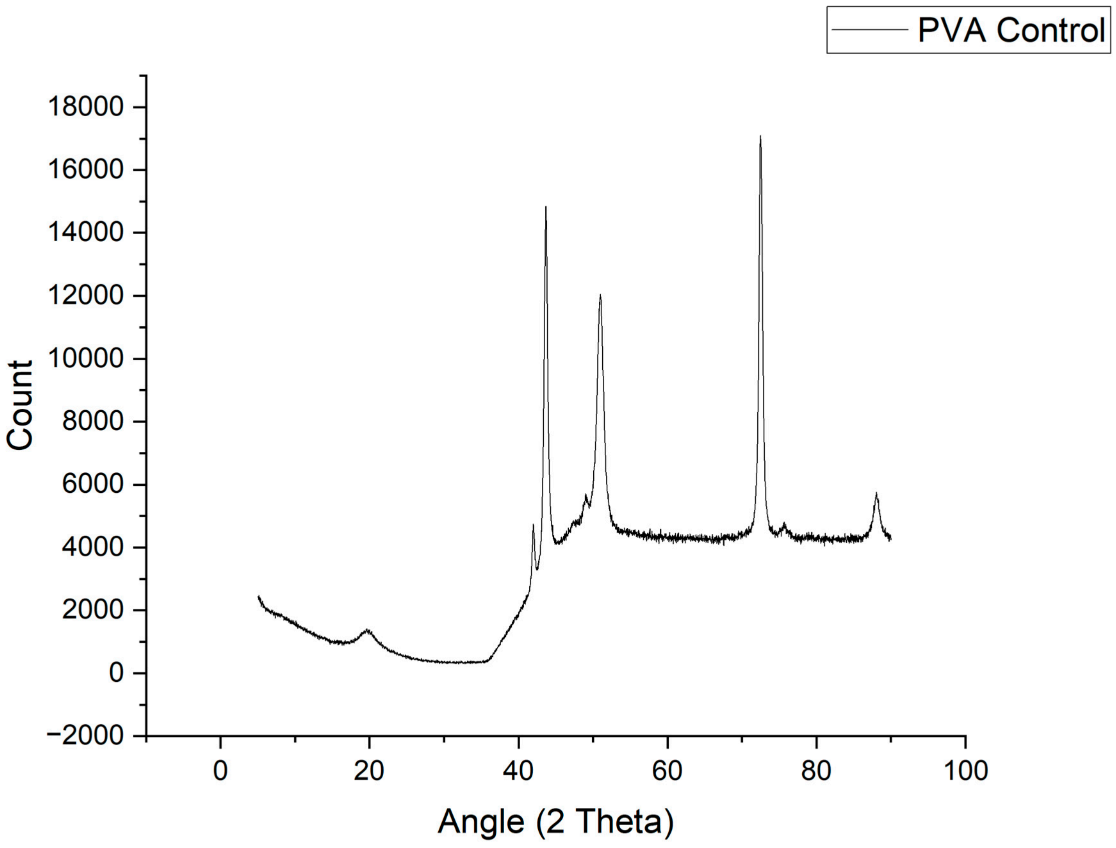

Figure S25: X-Ray Diffraction spectra of PVA nanofibrous yarn sample

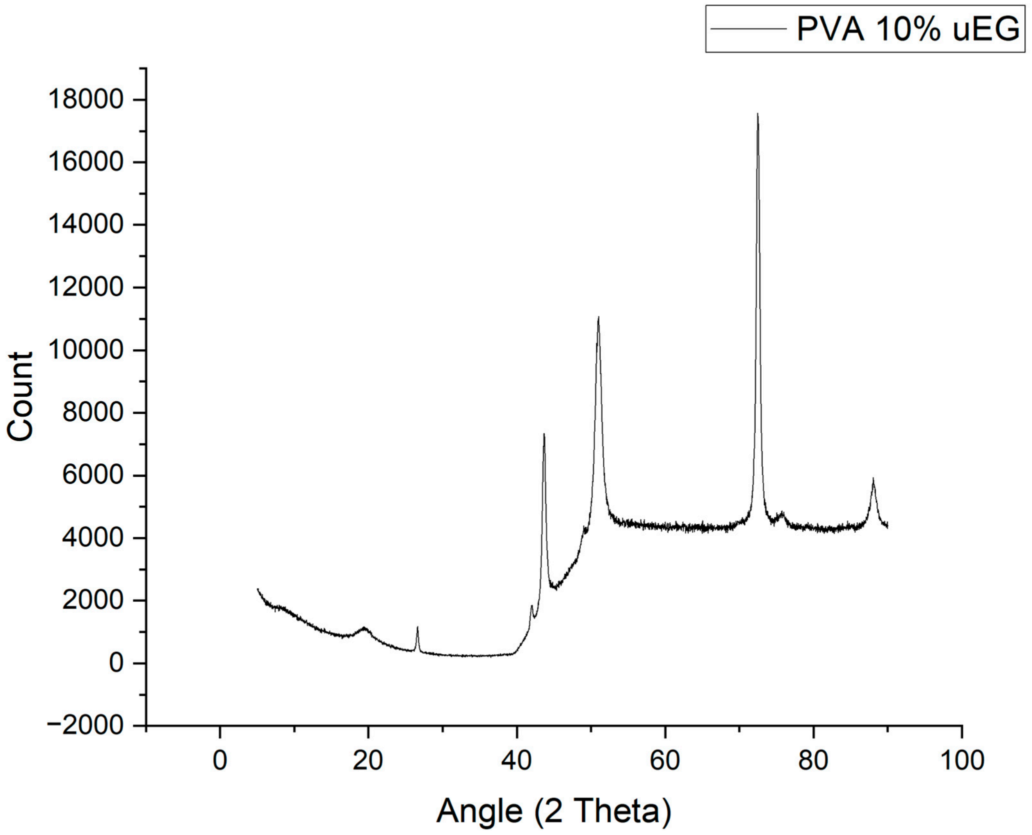

Figure S26: X-Ray Diffraction spectra of PVA 10% uEG nanofibrous yarn sample

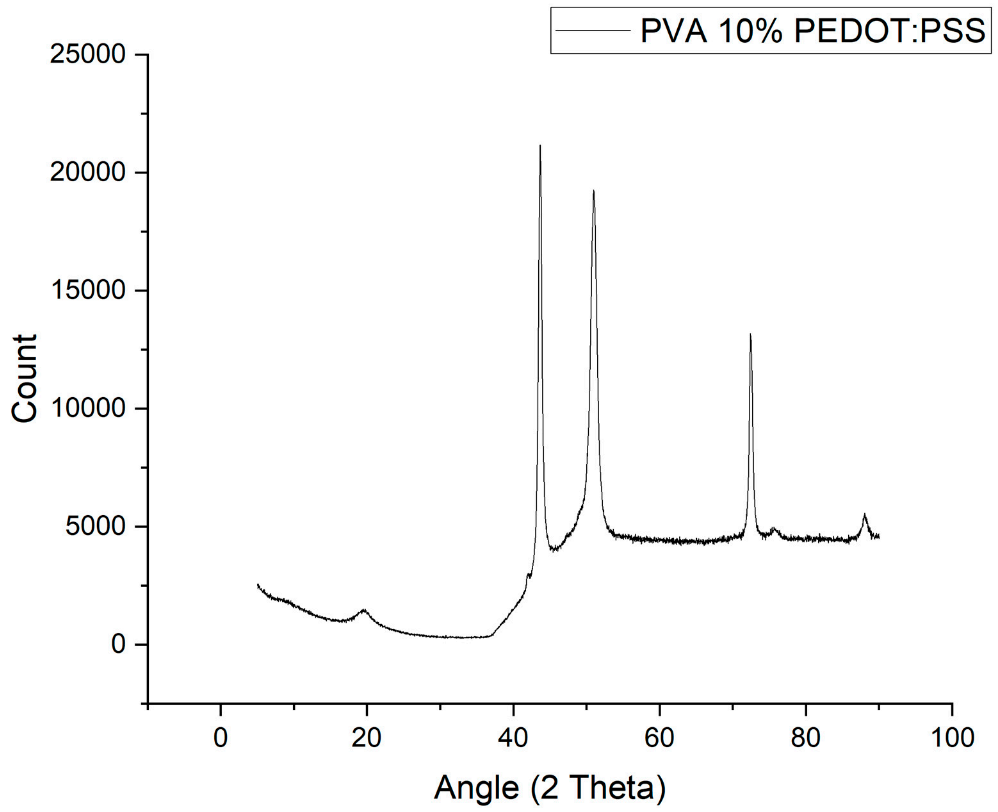

Figure S27: X-Ray Diffraction spectra of PVA 10% PEDOT:PSS nanofibrous yarn sample

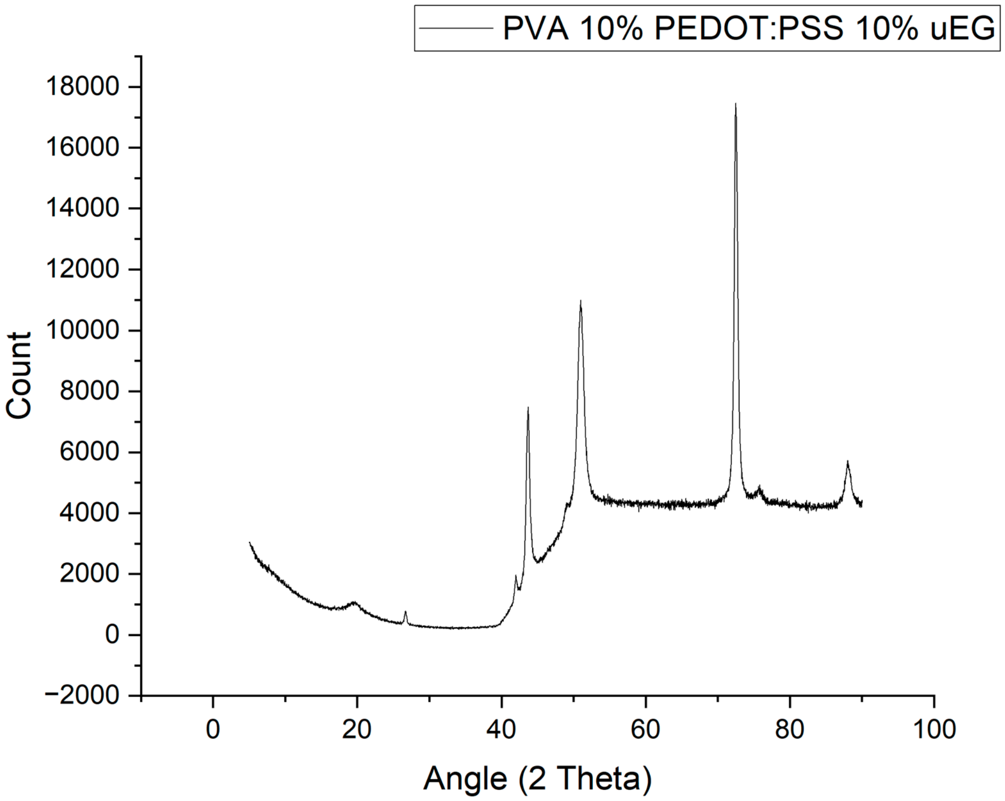

Figure S28: X-Ray Diffraction spectra of PVA 10% PEDOT:PSS 10% uEG nanofibrous yarn sample

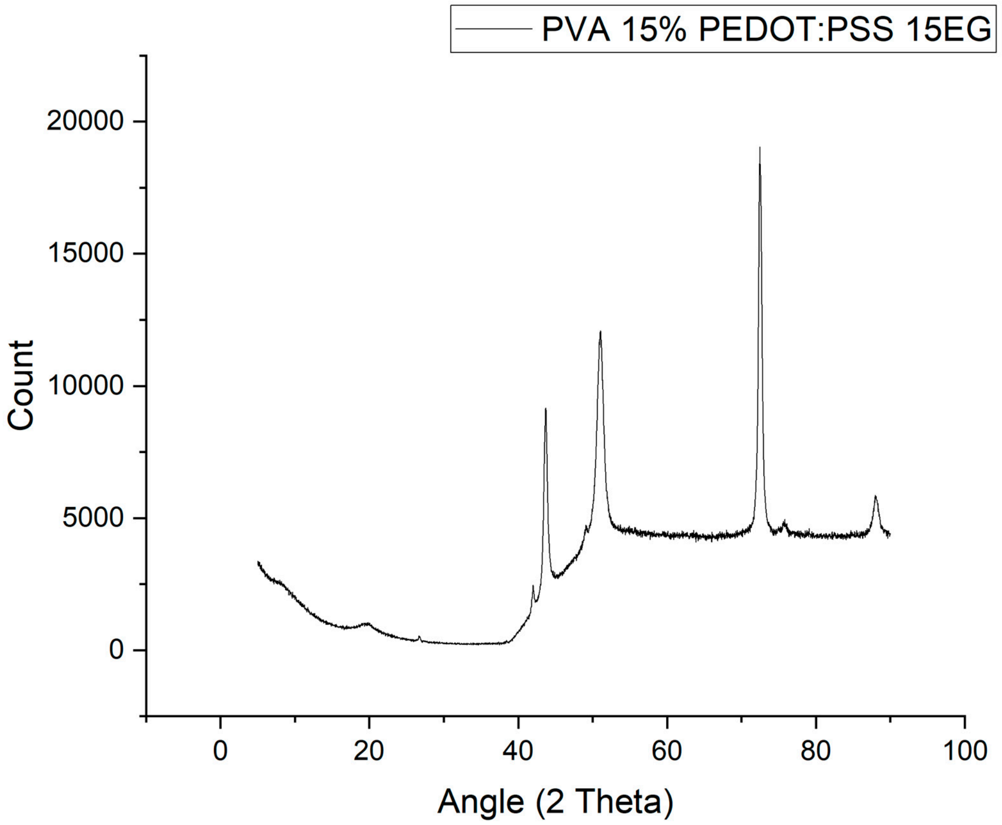

Figure S29: X-Ray Diffraction spectra of PVA 15% PEDOT:PSS 15% uEG nanofibrous yarn sample

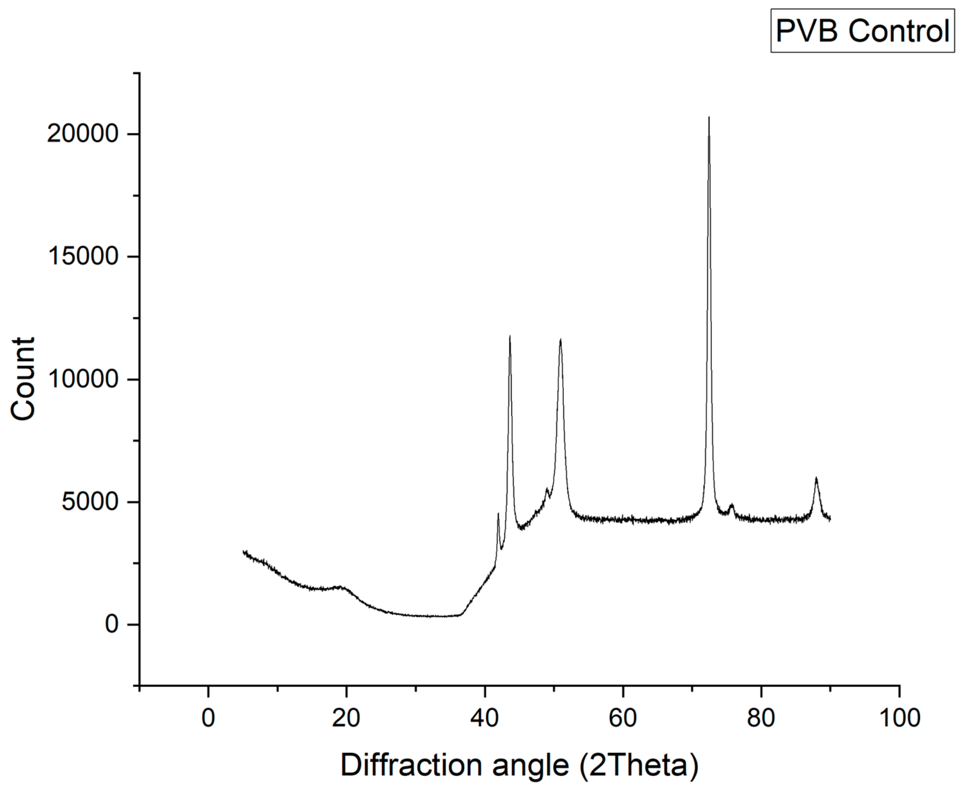

Figure S30: X-Ray Diffraction spectra of PVB nanofibrous yarn sample

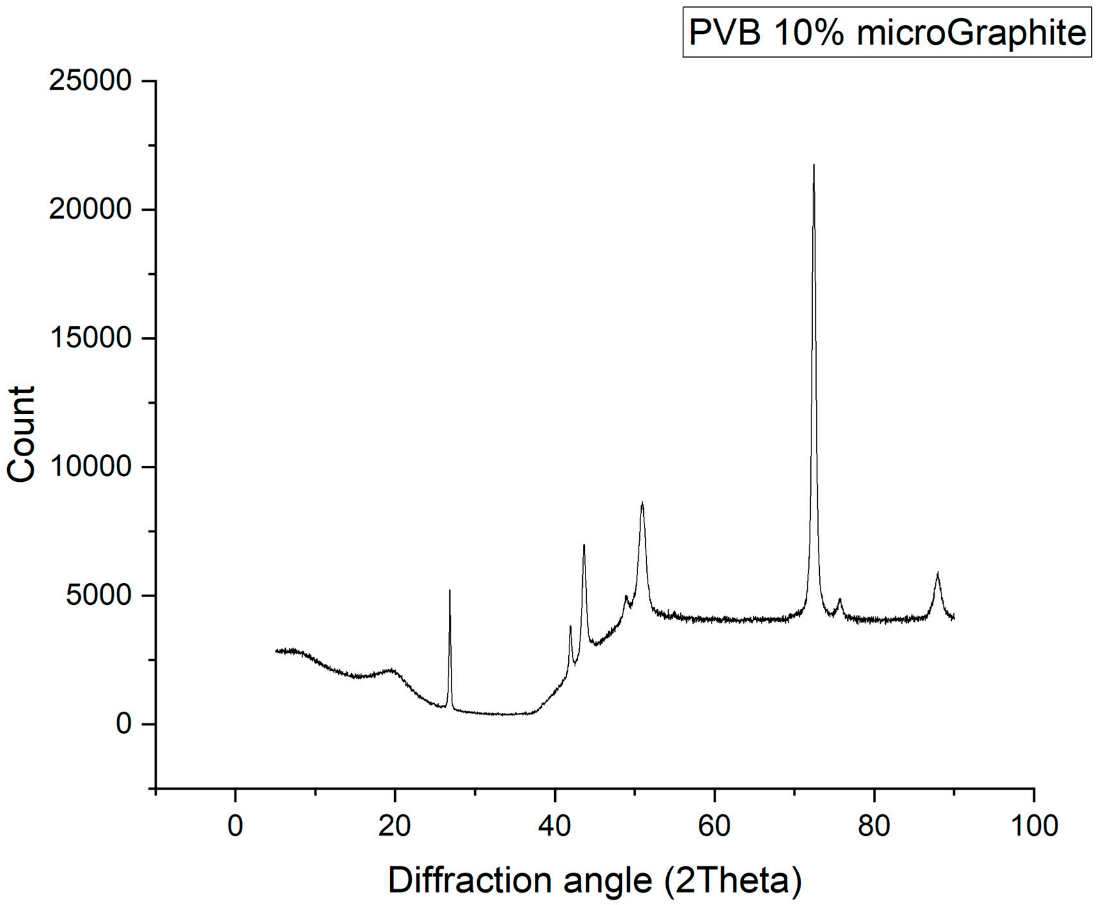

Figure S31: X-Ray Diffraction spectra of PVB 10% micro graphite nanofibrous yarn sample

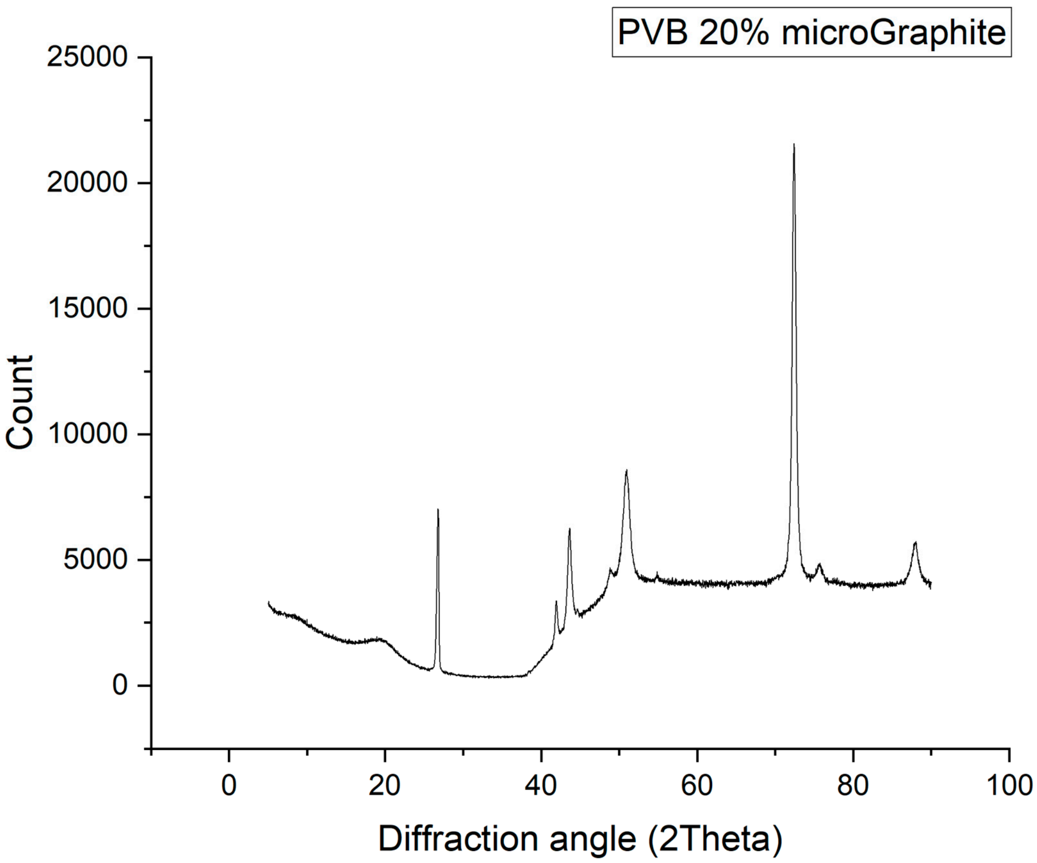

Figure S32: X-Ray Diffraction spectra of PVB 20% micro graphite nanofibrous yarn sample

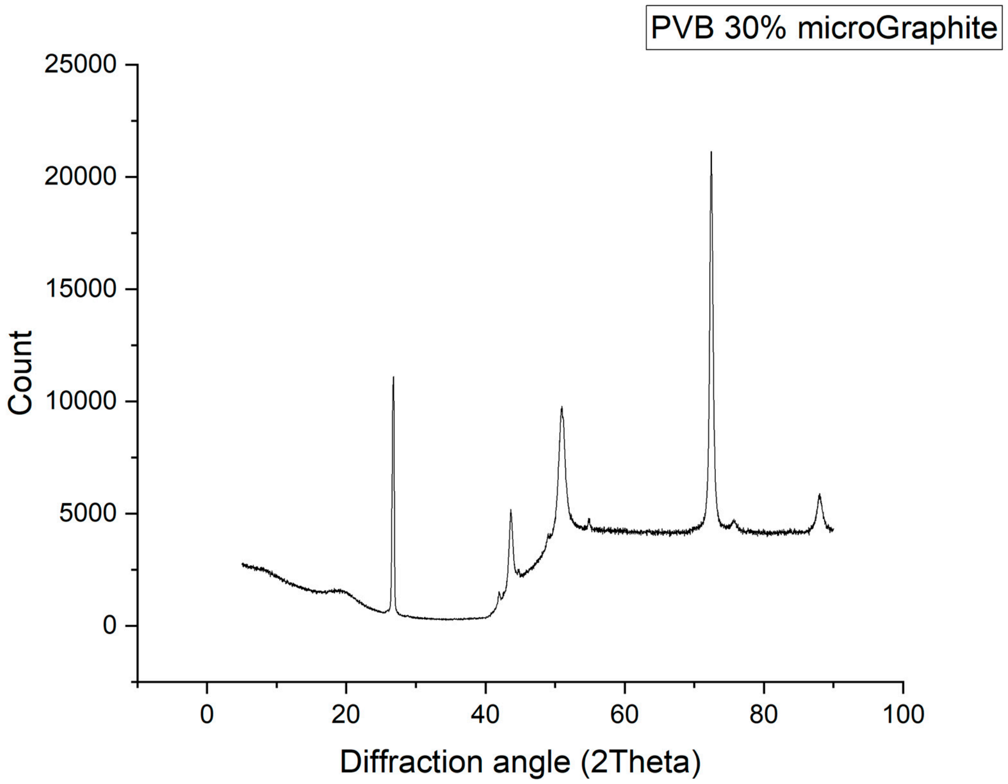

Figure S33: X-Ray Diffraction spectra of PVB 30% micro graphite nanofibrous yarn sample

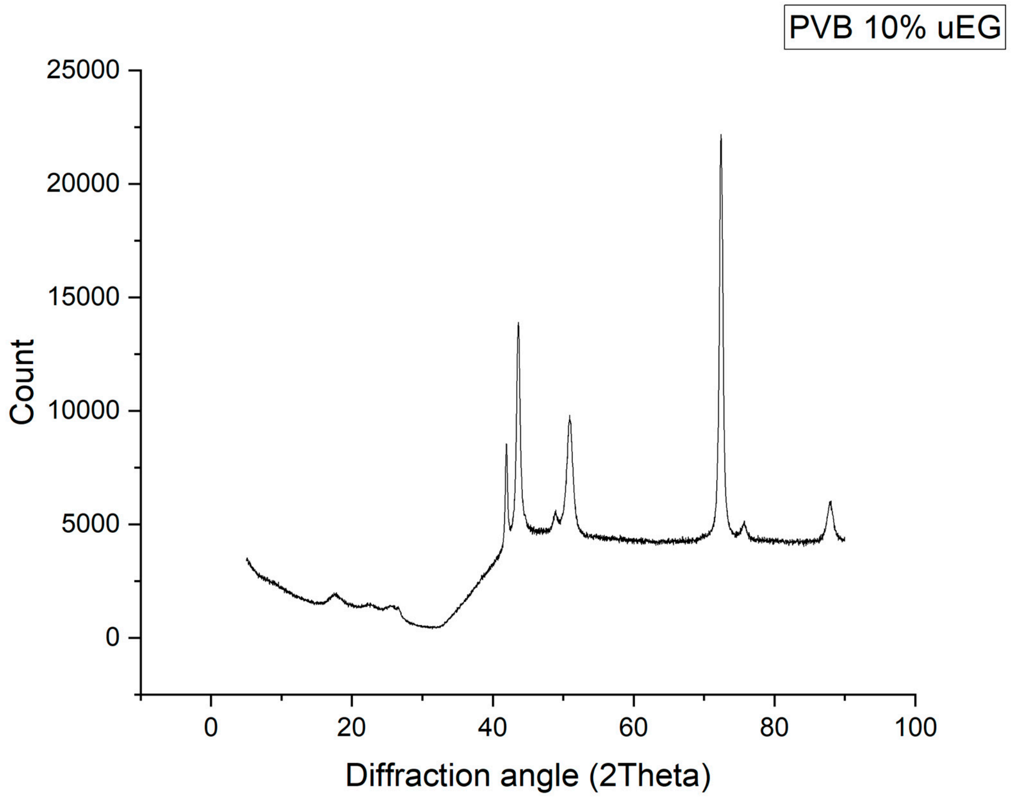

Figure S34: X-Ray Diffraction spectra of PVB 10% uEG nanofibrous yarn sample

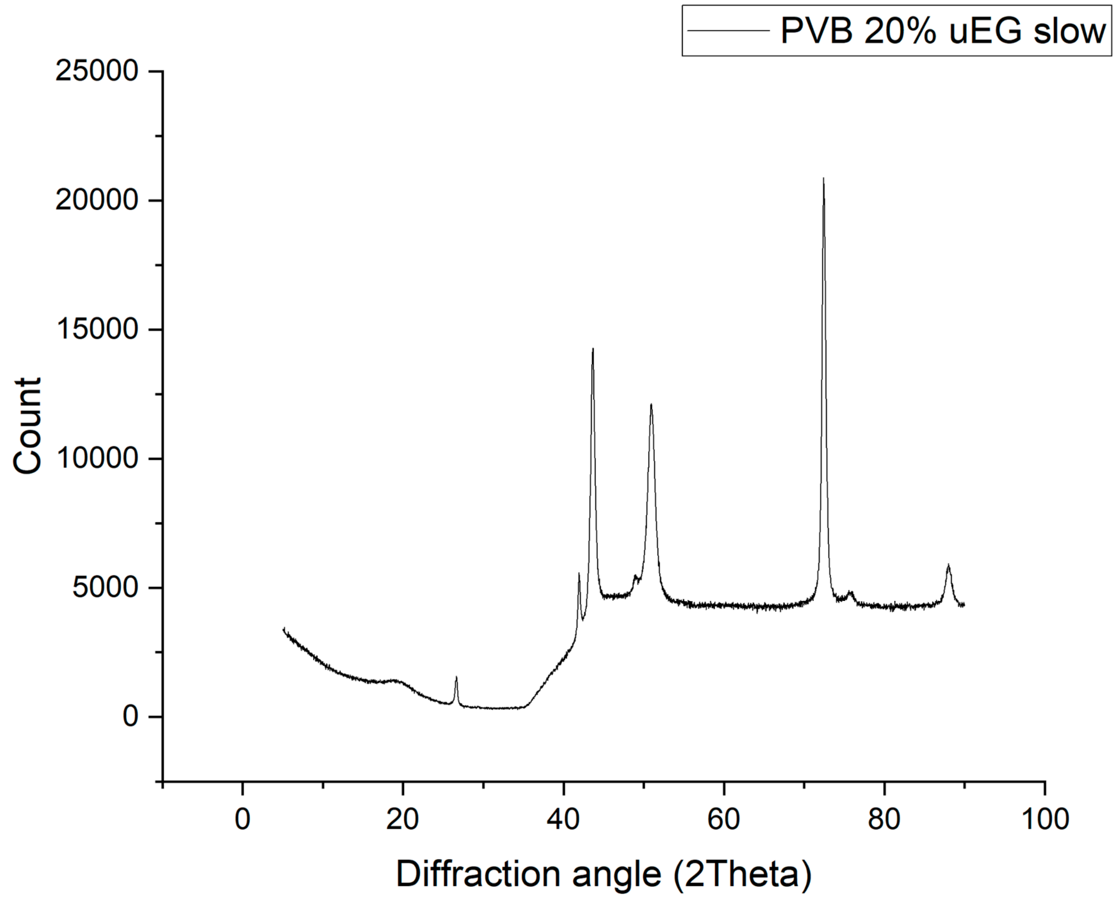

Figure S35: X-Ray Diffraction spectra of PVB 20% uEG slow nanofibrous yarn sample

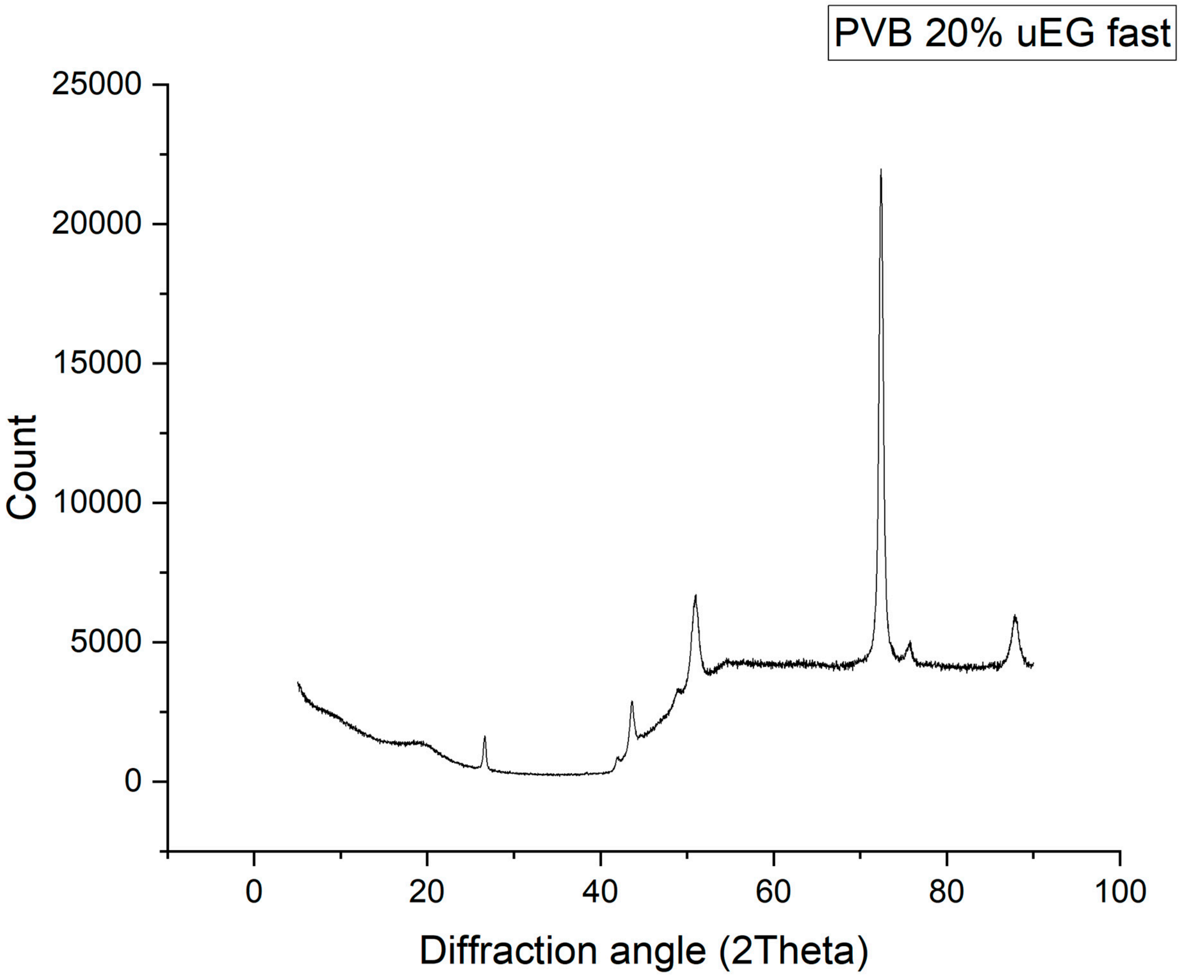

Figure S36: X-Ray Diffraction spectra of PVB 20% uEG fast nanofibrous yarn sample
